# Antibody afucosylation and enhanced autoreactive antibody response are associated with thyroid dysfunction during severe SARS-CoV-2 infection

**DOI:** 10.3389/fimmu.2026.1806272

**Published:** 2026-10-19

**Authors:** Elisa Pesce, Andrea Gobbini, Mauro Bombaci, Andrea Favalli, Sara Maioli, Stefano Artin Serapian, Noemi di Marzo, Luca Sorrentino, Mirco Toccafondi, Paola Gruarin, Andrea Lombardi, Mirco Armenti, Mariangela Lorenzo, Alessandra Bandera, Giorgio Colombo, Ilaria Muller, Renata M. Grifantini

**Affiliations:** 1 Istituto Nazionale di Genetica Molecolare (INGM) “Romeo ed Enrica Invernizzi”, Milan, Italy; 2 Department of Clinical Sciences and Community Health, Department of Excellence 2023-2027, University of Milan, Milan, Italy; 3 Department of Chemistry, University of Pavia, Pavia, Italy; 4 CheckmAb SrL, Milan, Italy; 5 Infectious Diseases Unit, Foundation IRCCS Ca’ Granda Ospedale Maggiore Policlinico, Milan, Italy; 6 Department of Pathophysiology and Transplantation, University of Milan, Milan, Italy; 7 Endocrinology Unit, Graves’ Orbitopathy Center, Fondazione IRCCS Ca’ Granda Ospedale Maggiore Policlinico, Milan, Italy

**Keywords:** afucosylation, autoantibodies, autoimmunity, SARS-CoV-2, thyroid disease, thyroiditis

## Abstract

**Introduction:**

SARS-CoV-2 infections causing severe COVID-19 are known to induce or exacerbate thyroid dysfunctions (TD), comprising autoimmune thyroiditis and subacute thyroiditis. Non-thyroidal illness syndrome is also common. Thyroid damage could result from direct viral infection and/or aberrant immuno-inflammatory responses. However, the specific immunological mechanisms contributing to TD in severe COVID-19 are not fully understood.

**Methods:**

We investigated the relationship between severe SARS-CoV-2 infection and TD by analyzing antibody autoreactivity and fucosylation level in 40 COVID-19 patients recruited during the 2020 pandemic wave. Using protein arrays, ELISA, and computational modeling, we compared patients with and without TD to 20 healthy controls and 29 Graves’ disease cases, focusing on the functional impact of the elicited antibodies.

**Results:**

We observed a significant shift in IgG autoreactivity, specifically targeting CD16A and KIR2DL1 in COVID-19 patients, both TD and not-TD, compared to Healthy Controls (HC) or other thyroid disease patients. Anti-Spike IgGs from COVID-19-TD patients exhibited a lower fucosylation state compared with those without TD and were associated with increased NK-cell activation and degranulation. Furthermore, patients’ sera showed increased binding to immortalized human thyroid epithelial cells. Computational analysis identified structural similarities between the SARS-CoV-2 Spike protein and thyroglobulin.

**Discussion:**

Thyroid damage in severe COVID-19 involves a complex interplay of different mechanisms. We proposed a potential role of lowly fucosylated anti-Spike antibodies, which may contribute to heightened NK cell-mediated activation via CD16A, potentially exacerbated by virus-induced autoreactivity. Altered IgG fucosylation and autoreactivity are associated with thyroid dysfunctions in severe COVID-19.

## 1 Introduction

COVID-19 (COronaVIrus Disease 2019) is a respiratory illness caused by the severe acute respiratory syndrome coronavirus 2 (SARS-CoV-2), which belongs to the coronavirus family (1). SARS-CoV-2 infection has been observed to result in a wide range of clinical manifestations, not only in the respiratory system, thus having the potential to cause tissue damage in various organs, in both the acute and severe phases of the infection. Although lung damage is the main cause of morbidity in COVID-19, involvement of multiple organs, including the thyroid gland, can also worsen the overall disease scenario. Different studies documented a link between anti-SARS-CoV-2 immune responses elicited during infection and onset of thyroid dysfunctions (TD) (2).

In our previous studies, we investigated thyroid-infiltrating T-lymphocytes in individuals who developed non-autoimmune atypical thyroiditis (AT) during severe COVID-19 (3–5). This clinical condition reflects the combination of non-thyroidal illness syndrome (NTIS), an aspecific alteration of thyroid hormones occurring during acute or chronic illnesses, with SARS-CoV-2-induced thyroiditis (6–8).

SARS-CoV-2 could impact the thyroid through multiple pathways: direct infection mediated by thyroidal ACE2 and TMPRSS2 expression (9), systemic inflammation via the cytokine storm (IL-1, IL-6, TNF-α) (10), and direct viral invasion from the respiratory tract (10). Additionally, molecular mimicry between the Spike protein and host antigens can trigger the production of pathogenic cross-reactive antibodies, leading to autoimmune-mediated thyroid dysfunctions. However, the immunological mechanisms contributing to the elicitation of TD are only partially elucidated (11).

In this study, we investigated the autoreactive antibody landscape in patients affected with TD during severe COVID-19. Protein array analysis revealed an aberrant immune response characterized by significantly elevated IgG levels against CD16A (FcγRIIIa) and KIR2DL1, receptors critical for NK cells and other innate immunity cells.

We also observed that COVID-19 patients with TD induce high levels of anti-Spike antibodies with low fucosylation state, which is associated with enhanced binding to FcγRIIIa and an increased NK degranulation, possibly leading to tissutal inflammation and pathogenetic effects. Antibodies of COVID-19 patients, including those with TD, showed a more efficient binding of immortalized human thyroid epithelial cells than patients with Graves’ disease (also known as autoimmune hyperthyroidism, since caused by autoantibodies stimulating the thyrotropin receptor) and healthy individuals. This evidence, combined with the lower fucosylation state of anti-Spike antibodies, could explain the local inflammatory state and tissutal damage observed in the thyroid gland during the acute phase of SARS-CoV-2 infection. Finally, computational analysis identified limited sequential similarities between highly immunogenic regions of thyroglobulin (Tg) and Spike protein. These observations, combined with reported molecular mimicry involving other thyroid proteins, suggest an additional mechanism that may contribute to thyroid dysfunctions.

Collectively, these observations highlight an intertwined event network leading to thyroid damage during severe COVID-19, and explaining the onset or worsening of TD.

## 2 Materials and methods

### 2.1 Patients’ selection

Patients with COVID-19 were recruited at Fondazione IRCCS Ca’ Granda Ospedale Maggiore Policlinico (Milan, Italy) and classified according to the WHO Ordinal Scale (12). The COVID-19 Group 1 included 12 patients with severe disease recruited within the Polimmune-COVID protocol, approved by the National Ethics Committee for COVID-19 trials at the Istituto Nazionale per le Malattie Infettive Lazzaro Spallanzani (approval #286_2021, February 2021; amended as #485_2021, December 2021) and subsequently by the Institutional Review Board Milano Area 2. The COVID-19 Group 2 included 28 consecutive patients hospitalized for severe COVID-19– (12) in the Department of Medicine - high intensity of care unit and enrolled in the study “Tiro-Covid-19” (Ethics Committee of Milano Area 2 ID 375_2020), with available stored sera collected at hospital admittance. SARS-CoV-2 infection was diagnosed by real-time reverse transcriptase polymerase chain reaction conducted on nasal and pharyngeal swab specimens or bronchoalveolar lavage fluid.

The Graves’ group included patients attending the Graves’ Orbitopathy Centre outpatient clinic of Fondazione IRCCS Ca’ Granda Ospedale Maggiore Policlinico in 2018, before the pandemic period, with stored sera available for analysis (Ethics Committee of Milano Area 2 ID 1036-2022).

Serum thyroid-stimulating hormone (TSH), free-thyroxine (FT4) and free-triiodothyronine (FT3) concentrations were measured by electrochemiluminescence immunoassay (Cobas^®^ e801, Roche Diagnostics, Germany). Reference intervals were 0.28 – 4.30 mIU/L for TSH, 8–17 ng/L for FT4 and 2–5 ng/L for FT3. Autoantibodies to thyroid peroxidase (TPOAb) and thyroglobulin (TgAb) were assessed by enzyme-linked immunosorbent assay (ThermoFisher, US), with normal reference ranges of <35 KIU/L (TPOAb) and <60 KIU/L (TgAb). Serum C reactive protein (CRP) concentrations were assessed by turbidimetry (Cobas^®^ c702, Roche Diagnostics, Germany), with normal reference ranges <0.5 mg/dL.

Age- and sex-matched healthy controls were recruited consecutively at Fondazione IRCCS Ca’ Granda Ospedale Maggiore Policlinico, prior to any SARS-CoV-2 exposure, and used to define baseline threshold in the different experiments (Ethics Committee of Milano Area 2, ID 708_2020).

None of the 28 patients with severe COVID-19 and available thyroid data (Group 2) had been vaccinated, since hospitalized from September 2020 to November 2020, before the availability of COVID-19 vaccines. Similarly, the 29 controls with Graves’ disease had not been vaccinated for COVID-19, since they had been sampled in 2018 before the SARS-CoV-2 pandemic. The prevalence of pre-existing hypothyroidism or thyroid autoimmunity in the COVID-19 cohort group 2 (N = 28) was 14%, in line with the prevalence observed in the general population (13).

### 2.2 Human protein array and serum profiling assays

HuProt arrays version 4.0 developed by CDI Laboratories were used. Incubation was performed according to the manufacturer’s instructions. The arrays were blocked using blocking buffer (2% BSA in PBS buffer with 0.05% Tween 20) at 4 °C overnight. Each serum sample was diluted at 1:500 in blocking buffer and incubated at room temperature (RT) for 2 h with gentle rocking. After 3 x 10 min washes with TPBS (PBS buffer with 0.05% Tween 20), the arrays were incubated for 2 h at RT with Alexa 647-conjugated goat α-human IgG (Thermo Fisher Scientific, USA) (1:800 in blocking buffer) in the dark with gentle rocking. The arrays were then washed two times in TPBS, two times in 0.1 x PBS, and finally one time in milliQ sterile water. The slides were finally dried at 30 °C under nitrogen and scanned using a ScanArray Gx PLUS (PerkinElmer, Shelton, CT). 16-bit images were generated with ScanArrayTM software at a resolution of 10 μm per pixel and analyzed using ImaGene 9.0 software (Biodiscovery Inc., Hawthorne, CA). A 635 nm laser was used to excite the Alexa-647 dye. See **Supplementary Methods** for analysis details.

### 2.3 Selection of candidate proteins

Autoantibodies were considered “positive” i) if MFI value of patients with COVID-19 for each protein was higher than the average MFI + 2 standard deviation (SD) values of HC sera, and ii) if recognition frequency was > 50% in the COVID-19 group and 0% in HC. Prioritization by protein localization is based on DeepLoc V1.0.0 and biomart.

### 2.4 NK purification from buffy coats

NK cells were isolated from the buffy coat of healthy donors. The buffy coat blood fraction was diluted 1:4 in PBS, and total PBMCs were obtained by density-gradient separation using Ficoll-Paque Plus (GE Healthcare, see supplementary). Cells were then pelleted, counted, and then used for NK-cell purification following the manufacturer’s instructions of Miltenyi NK Cell Isolation Kit (130-092-657) through negative selection. Following a 20-minute centrifugation at 800 × g with no brake, the PBMC layer was collected, washed with PBS, and centrifuged at 1400 rpm for 10 minutes. A second PBS wash followed by a low-speed centrifugation at 1000 rpm for 10 minutes was performed to remove platelets. Red blood cells were lysed for 5 minutes in the dark using BD Pharm Lyse (BD Biosciences, 555899).

### 2.5 Natural killer cell activation analysis by flow cytometry

NK cells were co-cultured with irradiated PBMCs at a 1:1 ratio in the presence of patient sera (1:10) for 4 hours, with Brefeldin A (10 μg/ml) added during the final 2 hours of incubation. As positive control cells were stimulated for 4 hours with 0.2 μM phorbol 12-myristate 13-acetate and ionomycin (1 μg/ml) in culture medium at 37 °C.

For the detection of cell surface proteins, cells were incubated with the indicated antibodies for 30 minutes at room temperature. After washing with MACS buffer (PBS containing 0.5% fetal bovine serum and 2 mM EDTA), cells were fixed for 20 minutes at 4 °C using CYTOfix/Cytoperm (BD). To detect intracellular proteins, cells were permeabilized and stained in Permeabilization Reagent supplemented with antibodies for 30 min at 4 °C. All incubation steps were performed in the dark.

### 2.6 ELISA

Serum IgG specific for the indicated recombinant antigens were quantified by ELISA. MaxiSorp 96-well ELISA plates (Cat. 44–2404, Thermo Fisher) were coated with 5 μg/ml of each recombinant protein diluted in phosphate-buffered saline (PBS), pH 7.4 overnight at 4 °C. ELISA plates were washed three times with 200 μl PBS containing 0.05% (v/v) Tween-20 (PBST). Plates were blocked with 200 μl of 10% BSA in PBST for 1 h at 37 °C, then incubated with diluted sera (1:50) in 1% BSA PBS for 1 hour at 37 °C. Plates were washed three times with 200 μl PBST and probed with 1:2000 HRP-conjugated α-human IgG secondary antibody for 45 minutes at RT. Finally, after three washes, to detect bound antibodies, 100 μl/well of development reagent containing 3, 3′, 5, 5′-Tetramethylbenzidine (TMB) were added. Color development was quenched after 3–5 minutes via addition of 100 μl/well 1 M sulfuric acid, and absorbance at 450 nm was measured with the Infinite F200 PRO plate reader (Tecan). Background-subtracted absorbance values were expressed as signal of the analyzed sera normalized to the internal control of each plate (S/Co).

For CD16A sera recognition, plates were coated with recombinant CD16A and blocked with 200 μl of 10% BSA. Plates were pre-incubated with commercial afucosylated mAb (Biosynth CLA1499) at saturating concentration 20 μg/ml in PBS 1% BSA for 1 hour at room temperature and then incubated with diluted sera (1:50) in 1% BSA PBS for 1 hour at 37 °C. The subsequent steps of the assay followed the standard protocol for detection.

### 2.7 Analysis of the antibody fucosylation

*Purification of SARS-CoV-2-Spike specific IgG from serum.* MaxiSorp 96-well ELISA plates (Cat. 44–2404, Thermo Fisher) were coated with 5 μg/ml of rec-SARS-CoV-2-Spike protein (50 µl/well) diluted in PBS, overnight at 4 °C. ELISA plates were then washed three times with 200 μl/well TBS containing 0.05% (v/v) Tween-20 (TBST). Plates were blocked with 200 μl of SuperBlock Blocking Buffer (Thermo Scientific, Catalog number 37515) for 30 minutes at 37 °C, then were incubated with patient sera diluted 1:10 in TBS (50 µl/well) 1 hour at 37 °C. Plates were washed three times with 200 μl TBST. To elute bound IgG, 100mM citric acid (pH3, 50 µl/well) was added and incubated for 5 min on an orbital shaker at room temperature (RT). To neutralize the acid solution, 6-10 µl/well of 1M Tris (pH9) was added and the pH was tested using pH indicator strips. The eluates were either directly processed or stored at 4 °C.

#### 2.7.1 Quantitation of antigen-specific IgG concentration

In order to determine the relative SARS-CoV-2-Spike specific IgG concentration of the generated eluates, SARS-CoV-2-Spike-ELISA was performed. In brief, MaxiSorp 96-well ELISA plates were coated with 5 μg/ml of rec- SARS-CoV-2-Spike protein (50 µl/well) diluted in PBS and were incubated overnight at 4 °C. After three washes with TBST, ELISA plates were blocked with 200 μl of SuperBlock Blocking Buffer for 30 minutes at 37 °C, then were incubated with SARS-CoV-2-Spike specific IgG (50 μl/well) for 1 hour at 37 °C. ELISA plates were washed three times with 200 μl TBST and were incubated with anti-Human IgG-HRP 1:2000 for 30 minutes at RT. Finally, after three washes, 100 μl/well of TMB-ELISA Substrate Solution was added. Color development was stopped after 3–5 minutes by addition of 100 μl/well 1 M sulfuric acid, and absorbance at 450 nm was measured with the Infinite F200 PRO plate reader (Tecan). Using Prism, the absorbance detected for each sample of purified IgG specific for SARS-CoV-2-Spike was interpolated with a concentration curve generated with the commercial SARS-CoV-2-Spike antibody.

#### 2.7.2 Analysis of relative SARS-CoV-2-spike specific IgG-Fc fucosylation

Fucosylation levels of S-specific IgG were measured using a lectin-based ELISA assay. In brief, 96-well MaxiSorp plate was coated with 50 µl/well of eluted SARS-CoV-2-Spike specific IgG overnight at 4 °C. ELISA plate was washed three times with 200 μl TBST. Unspecific binding sites were blocked adding 200 µl/well Carbo-free blocking solution (VectorLab.) for 1 hour at RT. After three washes with TBST, 100 µl/well of 4 µg/ml (1:125) conjugated Lectin-HRP from *Aleuria Aurantia* (AAL) diluted in carbohydrate-free blocking solution was added and incubated for 45 minutes at RT. To remove unbound lectin, the plate was washed with 200 µl/well TBST and relative fucosylation was detected by the addition of 100 µl/well TMB chromogenic substrate for AAL-bound peroxidase. Color development was quenched after 3–5 minutes via addition of 100 μl/well 1 M H_2_SO_4_. Thus, the absorbance was quantified at OD450nm using Infinite F200 PRO plate reader and the relative fucosylation for each generated pool-eluate was calculated by normalizing OD450nm (fucosylation) to its respective relative antigen-specific IgG amount.

### 2.8 Statistical analysis

*Protein Array.* The demographic and clinical characteristics of participants were summarized using descriptive statistics. Normalized data and statistical analysis of proteins spotted into the HuProt Array were analyzed by R as mentioned in the previous section and in the **Supplementary Materials**.

For the other immunological assays statistical analysis is performed with Graphpad Prism and details regarding the adopted test are reported under each figure.

## 3 Results

### 3.1 Study design

In this study we recruited 40 patients who experienced COVID-19 in 2020 (COVID-19 patients), before the vaccination campaign. Within these patients we distinguished 36 individuals who were hospitalized for severe COVID-19 and 4 individuals with a mild form of disease. In these individuals, the IgG response against wt SARS-CoV-2-S protein was assessed by ELISA. As shown in **Figure 1**, for 12 of the 40 individuals (COVID-19 group 1), thyroid tests were not included in the clinical assessment at diagnosis. For 28 individuals (COVID-19 Group 2), a more accurate clinical routine was done also including thyroid history and biochemical analysis at hospital admission of serum concentrations of thyroid stimulating hormone (TSH), free-thyroxine (FT4) and tri-iodothyronine (FT3), and detection of autoantibodies to thyroid peroxidase (TPOAb) and thyroglobulin (TgAb). This analysis identified 11 individuals of the COVID-19 Group 2 with thyroid dysfunctions (COVID-19-TD), mainly due to transient COVID-19-related AT as previously described (3, 4). Serum or plasma samples were collected from both COVID-19 groups (for Group 1 at 19–51 days after positive swab; for Group 2, during hospitalization for COVID-19, at 1–10 days from positive swab). For comparative analysis we also included 29 individuals with Graves’ disease and orbitopathy (Graves’ group) and 20 healthy controls (HC), both collected before COVID-19 pandemic. The demographic and clinical characteristics of all patients are shown in **Tabless S1**, **S2**.

**Figure 1:**
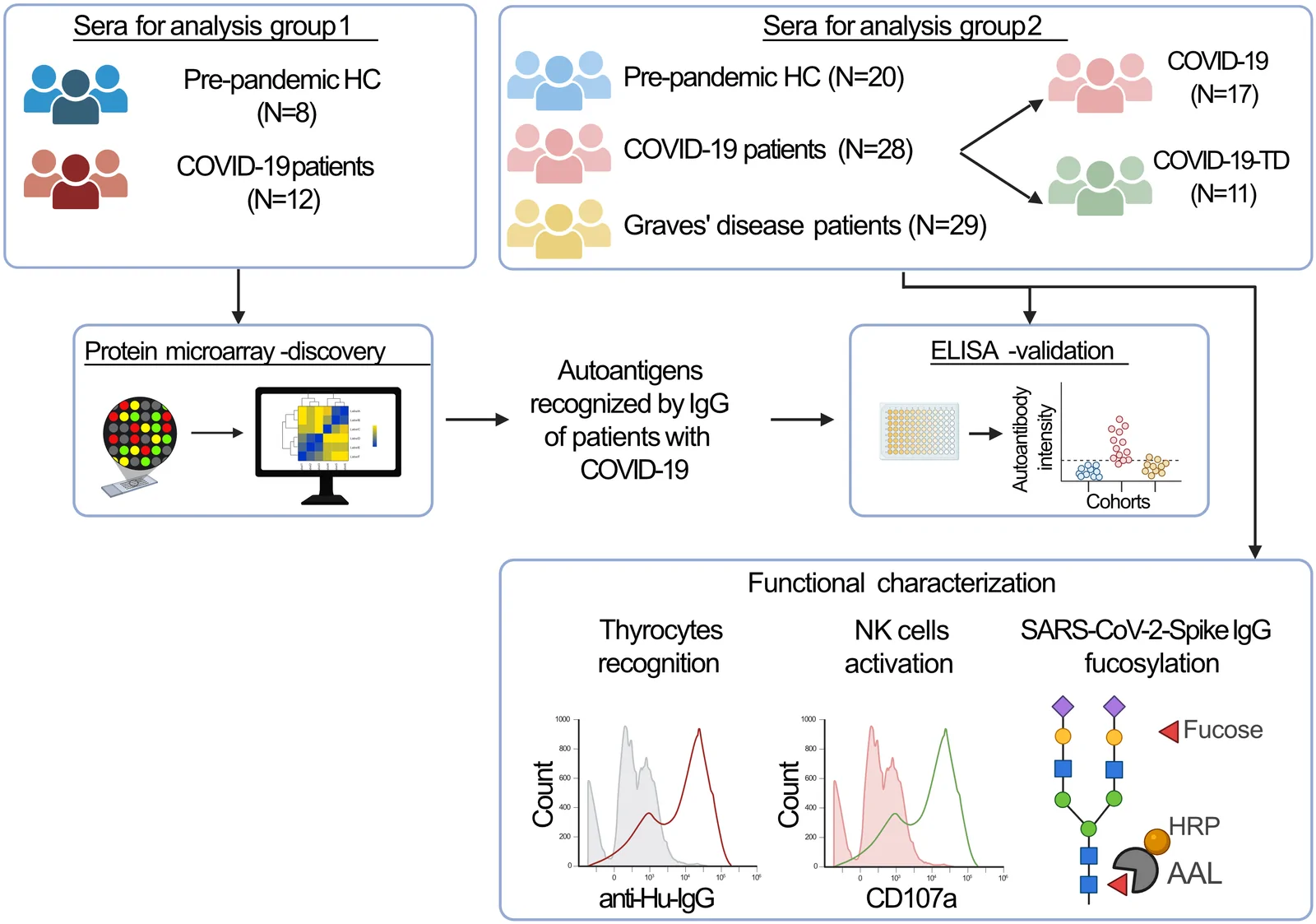
Study overview. Pre-pandemic healthy subjects (HC, n=8) and COVID-19 Group 1 (n=12) were screened via protein arrays to identify IgG-recognized autoantigens. Validation involved a second COVID-19 cohort (Group 2, n=28), stratified into those with thyroid disease (COVID-19-TD, n=11) and those without (n=17), alongside pre-pandemic Graves’ disease (n=29) and HC (n=20) controls. Functional characterization included: I) ELISA for autoantigen reactivity; II) lectin-based ELISA to quantify Spike-specific IgG fucosylation; and III) flow cytometry to assess NK cell activation (CD107a expression) and immortalized human thyroid epithelial cells recognition. (ALL: Lectin-HRP from Aleuria Aurantia, HRP: Horseradish Peroxidase).

In our experimental approach, we first investigated the autoreactive antibody response in serum samples of COVID-19 group 1 and 8 HC using a protein array platform and identified proteins showing differential reactivity in the two groups. We then confirmed the altered antibody response against the selected proteins by ELISA, using sera of COVID-19 group 2, as compared to sera of Graves’ and HC groups.

Sera of COVID-19 group 2 were then used in a series of biochemical and immunological analyses. Specifically, we focused on the differences between antibodies elicited in COVID-19 individuals with TD, compared to those who had only COVID-19, to understand peculiarities and potential pathogenicity of elicited antibodies that could explain the onset of AT during SARS-CoV-2 infection (see scheme in **Figure 1**).

### 3.2 Patients with COVID-19 develop autoantibodies against human proteins

Autoantibodies have been detected in COVID-19 patients, but their clinical significance remains poorly defined (14–16). To characterize the SARS-CoV-2-induced IgG autoreactive response, we screened serum samples from COVID-19 Group 1 and eight HC subjects using a high-density human protein microarray covering 80% of the proteome (**Supplementary Table S1**; **Figure 1**).

Overall, sera of COVID-19 group 1 showed a higher autoreactivity than HC sera (**Figure 2A**), with a differential IgG binding to 358 putative autoantigens, which was assessed by differences in mean fluorescence intensity (MFI) values (**Figure 2**; **Supplementary Table S3**). Among them, we focused on those specifically recognized by COVID-19 sera (recognized by at least 50% of COVID-19 patients, 0% of HC) and localized on the plasma cell membrane or secreted (i.e. proteins potentially accessible for binding to autoantibodies, **Figure 2B**). Five proteins involved in the immune response were identified as putative antigens, including IFNγ, IL1RN, FCGR3A (CD16A), KIR2DL1 and CRLF3, among which IFNγ and IL1RN have already been correlated with increased severity of COVID-19 (17, 18). CD16A and KIR2DL1 are two receptors highly expressed in innate immunity cells (19, 20), such as NK cells. CRLF3, has been reported as general neuro- or tissue-protective receptor for Epo-like cytokines across species (21).

**Figure 2:**
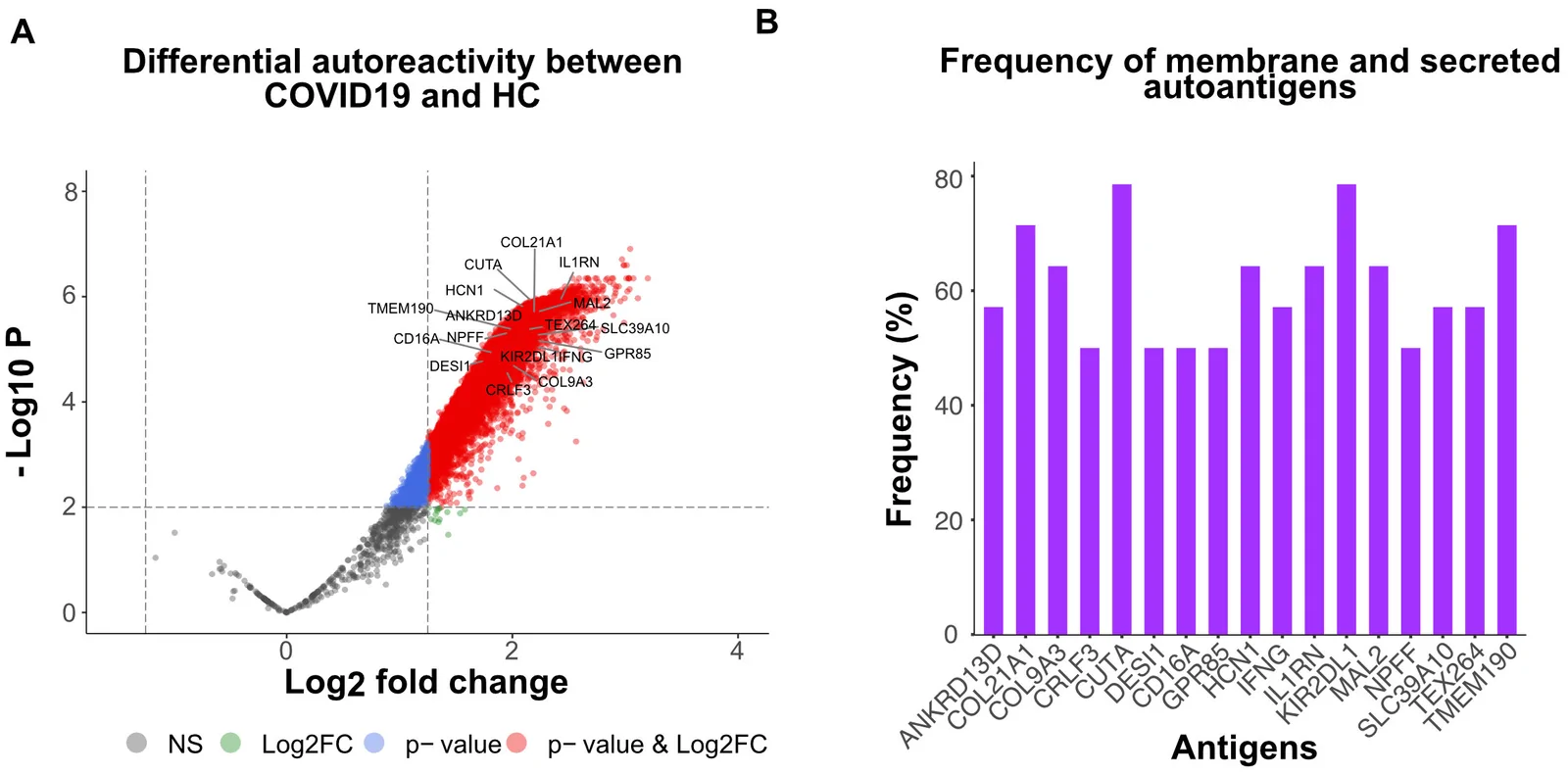
Autoreactive antigens recognized by COVID-19 patients’ sera. **(A)** Volcano plot showing aberrant reactivity of sera from COVID-19 group 1 patients as measured by protein array (HuProt v4) compared to pre-pandemic healthy controls (HC). y axis reports log10 p-values (-Log10 P) while x axis reports log2 fold change. Selected highly reactive proteins are reported. **(B)** Barplot showing frequency of COVID-19 sera showing a higher reactivity for the selected proteins than HC sera.

### 3.3 During COVID-19, autoantibodies are elicited against CD16A and KIR2DL1

We focused our analysis on the proteins involved in the immune system, with specific interest for CD16A, KIR2DL1 and CRLF3, which are known to be involved in inflammation. To confirm their altered reactivity during COVID-19, we analysed them by ELISA using sera of the COVID-19 group 2 (28 sera) and 20 HC (**Figure 1**; **Supplementary Table S1**). Compared with levels in pre-pandemic HCs, levels of IgGs against CD16A and KIR2DL1 antigens were significantly elevated in COVID-19 group 2 subjects (p<0.0001), while CRLF3 did not show differential reactivities (p>0.05) (**Figures 3A, B**). We then analysed separately the reactivity of COVID-19-TD and COVID-19 patients, also adding an independent cohort of patients with pre-pandemic Graves’ disease (n=29; **Supplementary Table S1**). COVID-19 individuals showed higher anti-CD16A and anti-KIR2DL1 IgG levels than Graves’ and HC subjects, irrespective of the presence of thyroid dysfunctions (**Figures 3C, D**).

**Figure 3:**
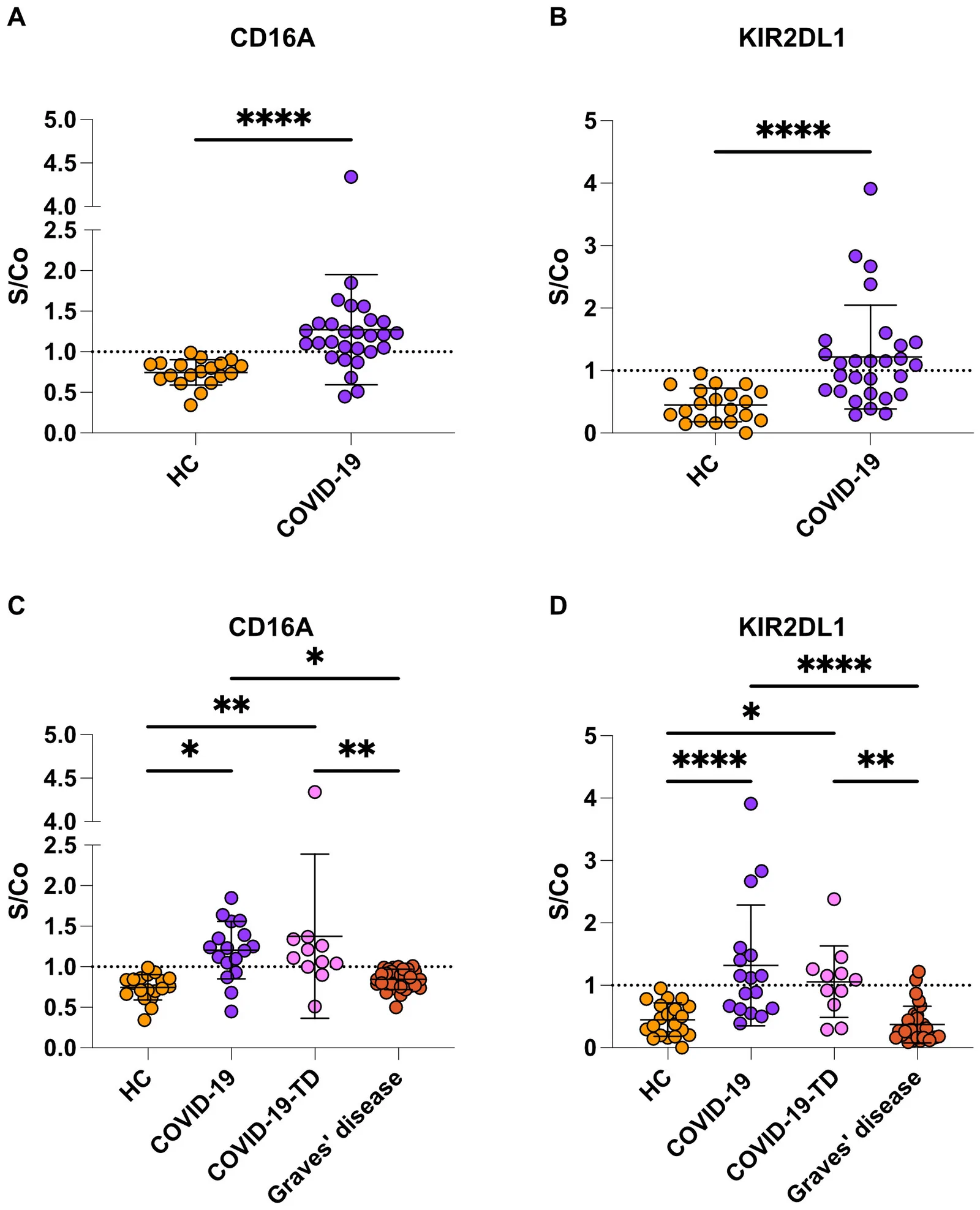
CD16A and KIR2DL1 display a higher immunoreactivity with sera of COVID-19 and COVID-19-TD patients than control groups. ELISA quantification of CD16A and KIR2DL1 reactivity. **(A, B)** Comparison between COVID-19 Group 2 (n=28) and healthy controls (HC, n=20). **(C, D)** Stratified analysis of COVID-19 with thyroid dysfunctions (COVID-19-TD, n=11) and COVID-19 (n=17) versus pre-pandemic Graves’ (n=29) and HC cohorts. Results are expressed as Signal/Control (S/Co) ratios, with the dotted line indicating the positivity threshold (mean HC + 1 standard deviation). COVID-19 groups displayed significantly higher immunoreactivity. Statistics: Mann-Whitney U and one-way ANOVA tests (∗p<0.05; ∗∗p<0.01; ∗∗∗∗p<0.0001). The ability of patients’ IgG to recognize CD16A via the Fab fragment, beside the canonical Fc interaction, was inferred by competitive ELISA using commercial afucosylated mAb (**Supplementary Figure 7**).

### 3.4 Sera from individuals with thyroid dysfunctions during COVID-19 are able to bind CD16A expressed by NK cells and induce their degranulation activity

CD16A and KIR2DL1, recognized by sera of the COVID-19 group 2, are highly expressed by NK cells, macrophages, monocytes and neutrophils and have effector function (22, 23). In particular, CD16A is a well known activating receptor of the Fc portion of antibodies, particularly IgG (24). We hypothesized that the altered antibody reactivity for CD16A occurring during severe COVID-19 may activate NK cell function, potentially contributing to an inflammatory response.

To address this point, we selected the serum samples most reactive to CD16A and with SARS-CoV-2-Spike protein. Whole serum was used in this experiment, rather than purified antibodies, to reflect a physiological context.

As a trend, we noted that anti-Spike antibody reactivity was higher in the COVID-19-TD than in the COVID-19 cohort (**Supplementary Figure S1**). Selected samples were analyzed for the ability to bind CD16A on NK cells by competitive flow cytometry, using increasing concentrations of recombinant CD16A as competitor. Data showed that sera from COVID-19-TD specifically bound CD16A on the NK cell membrane (**Supplementary Figures S2A-D**).

Furthermore, we assessed the influence of sera on NK activation and cytotoxicity by measuring surface expression of CD107a (LAMP-1), a marker of degranulation, on their CD16A^+^CD56^dim^ subset (**Supplementary Figure S3**). As a trend, sera from the COVID-19-TD cohort induced higher expression of CD107 on NK cells than sera from HC, Graves’ disease and COVID-19 group 2 with peaks in some patients. In particular, a higher percentage of sera (55%) from the TD groups induced the expression of CD107a, indicating an enhanced activation of NK-cell cytotoxic activity (**Figure 4A**).

**Figure 4:**
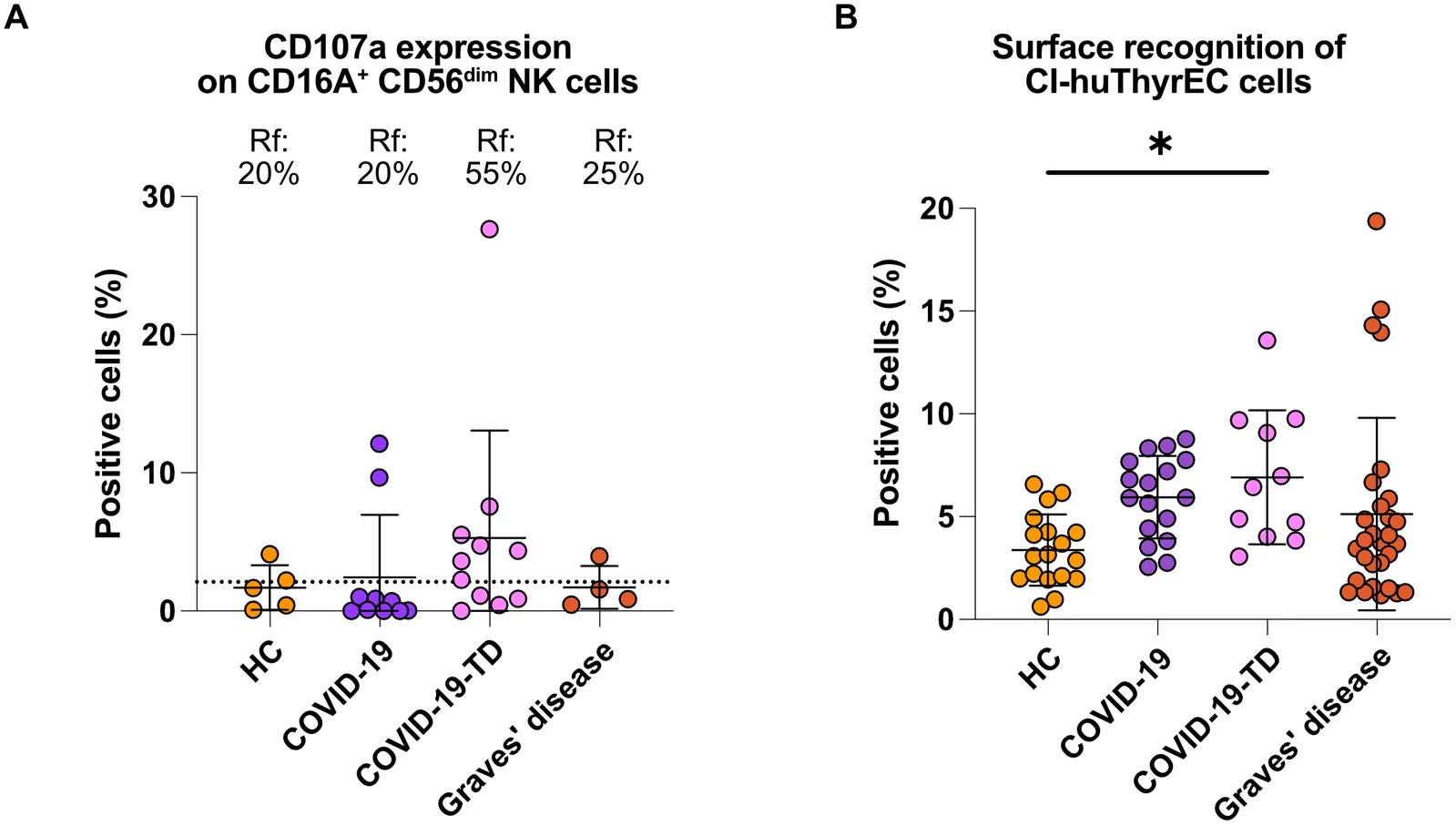
Sera effect on NK and thyrocyte cells. **(A)** Sera of individuals with thyroid disfunctions (TD) induce the accumulation of the cytotoxic granule membrane protein CD107a on the surface of NK cells. Flow cytometry analysis of CD107a expression on CD16A^+^CD56^dim^ NK cells (purified from buffy coats of 2 donors). The graph compares positive cells (%) after NK cells incubation with sera from healthy controls (HC, n=5), COVID-19 (n=10), COVID-19-TD (n=11), and Graves’ disease (n=4). The recognition frequency (Rf) denotes the percentage of sera exceeding the threshold (mean HC + 1 standard deviation), as average value obtained with buffy coats of two donors. Data indicates that COVID-19-TD sera significantly induce cytotoxic granule mobilization, suggesting enhanced NK cell activation compared to other groups. **(B)** Serum of individuals affected by COVID-19 binds to human thyrocytes. Surface recognition of CI-huThyrEC cells by HC (n=18), COVID-19 group 2 (n=17), COVID-19-TD (n=11) and Graves’ disease (n=29) sera measured by flow cytometry. Each individual serum (1:50 dilution) was incubated with 200, 000 cells. IgGs that bound to the thyrocyte plasma membrane were detected by anti-human IgG/Alexa Fluor 647 antibody and analysed by flow cytometry. Statistical analyses were performed using one-way ANOVA test. P value: *<0.05.

### 3.5 Sera from individuals with COVID-19 show a higher binding to human thyroid cells

Since protein array data highlighted a marked autoreactivity induced by COVID-19, which could cause potential damage to human cells, we exploited flow cytometry to investigate the ability of sera from COVID-19-TD patients to bind Human Thyroid Epithelial Cells (CI-huThyrEC), compared to those from patients with COVID-19 only, Graves’ disease or HC individuals. As a trend, sera from COVID-19 patients showed a better binding of thyrocytes if compared to HC, with some peaks in the COVID-19-TD group (**Figure 4B**).

As reported, some sera from individuals with Graves’ disease showed positive binding to thyrocytes, possibly due to the known presence of autoantibodies against specific autoantigens, such as thyrotropin receptor (TSHR) on the surface of thyrocytes. Such binding could also stimulate cells, leading to increased thyroid hormone production (25, 26).

Since thyroid cells physiologically express ACE-2, the main SARS-CoV-2 receptor on epithelial cells, we further investigated whether the binding of COVID-19-TD sera to CI-huThyrEC was also indirectly strengthened by the presence of anti-Spike antibodies. We incubated sera of COVID-19-TD and COVID-19 (with anti-Spike titers >3200) with CI-huThyrEC cells, with or without prior incubation with recombinant Spike (1 µg/ml). We showed that the presence of Spike further increased the binding of sera on the cell line, especially in the COVID-19-TD group (**Supplementary Figure S4**).

### 3.6 Anti-spike IgGs show lower level of fucose on the Fc core in COVID-19 patients with thyroid dysfunctions

IgG-Fc glycans are critical for antibody effector functions, as the absence of core fucose significantly enhances affinity for CD16A, on NK cells and macrophages. Given that severe COVID-19 is associated with reduced IgG fucosylation (27), we hypothesized that COVID-19-TD patients exhibit a distinct afucosylation profile. Using a lectin-based ELISA with *Aleuria aurantia* lectin (AAL) (28), we analyzed purified Spike-specific IgGs from COVID-19 Group 2. We observed significantly lower core fucosylation in the COVID-19-TD group compared to patients without thyroid complications (**Figure 5**), a finding further validated by lectin blotting (**Supplementary Figure S5**). Notably, total IgG fucosylation levels remained comparable between COVID-19-TD and healthy cohorts (**Supplementary Figure S6**), indicating that this reduced fucosylation is a distinct feature of Spike-specific IgGs.

**Figure 5:**
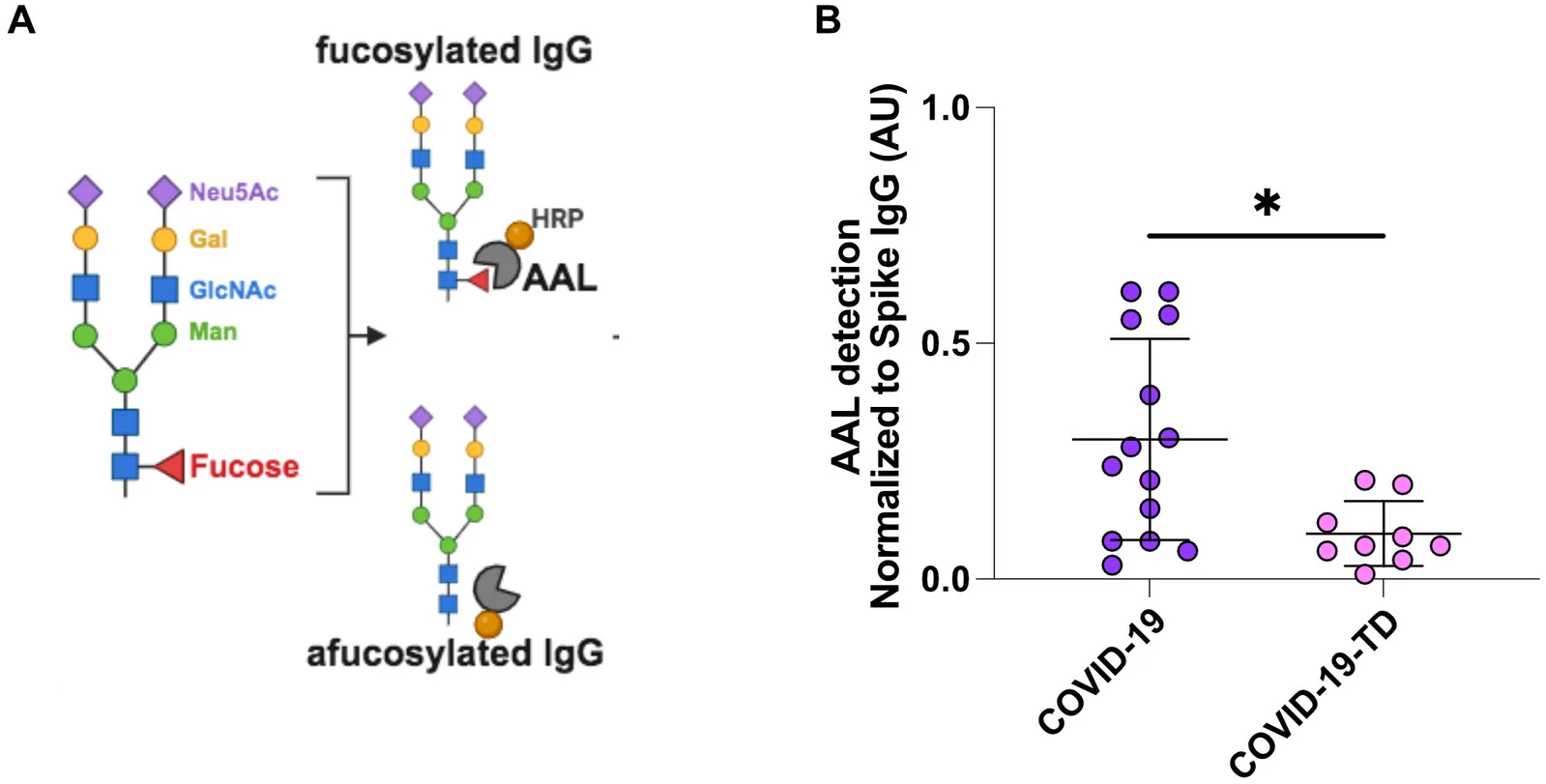
IgGs specific for SARS-CoV-2-Spike show a lower core fucosylation in COVID-19-TD patients. **(A)** Schematic of IgG-Fc core fucose and its detection via *Aleuria aurantia* lectin (AAL) binding. HRP: Horseradish Peroxidase. N-Acetylneuraminic acid (Neu5Ac), Mannose (Man), Galactose (Gal), N-Acetylglucosamine (GlcNAc). **(B)** Fucosylation levels of purified Spike-specific IgGs from COVID-19 (n=17) and COVID-19-TD (n=11) patients measured by lectin-ELISA. OD values (AAL-HRP) were normalized to antigen-specific IgG concentrations and represented by arbitrary unit (AU). Results demonstrate a significantly decreased fucosylation of Spike-specific IgGs in patients with thyroid complications (COVID-19-TD). Statistical analysis was performed using an unpaired t-test (∗p<0.05).

### 3.7 SARS-CoV-2 Spike protein shows regions of structural and sequential similarity with predicted thyroglobulin epitopes

To investigate potential mechanisms of structural mimicry, we assessed sequence homology between the SARS-CoV-2 Spike protein and a panel of proteins highly expressed in thyroid gland cells and known to play pivotal roles in thyroid function (**Supplementary Table S4**). Among the analyzed proteins, thyroid peroxidase (TPO) has already been reported by other studies as potentially involved in molecular mimicry with SARS-CoV-2 proteins. We prioritized Tg protein for further investigation, as it shares a moderate amino acid sequence identity with Spike (21.76%) and is a well-known autoantigen in thyroid disorders (TD), being a frequent target of autoantibodies in thyroid autoimmunity (29) whose possible relationship with COVID-19 has not yet been elucidated (30, 31).

Moreover, Tg emerged as one of the thyroid proteins with the highest homology to SARS-CoV-2 Spike protein for which it was easiest to reconstruct a viable human model. We based ourselves on this human model to predict potential epitope regions, using the Matrix of Low Coupling Energies (MLCE) method. The two largest potential epitopes detected on thyroglobulin spanned a major portion of each arm domain (the largest is shown in red in **Figure 6A**). We thus decided to isolate part of the arm domain from thyroglobulin protomer B and subject it to a structural similarity search against all proteins in the PDB using the DALI server (32). Intriguingly, our search predicted a degree of similarity between the arm domain of thyroglobulin and regions on the Spike proteins of both the human MERS-CoV and the Feline Infectious Peritonitis Virus, with *Z-*scores of 3.0 and 2.8 respectively (*Z-*scores ≥2.0 denote similarity). We show one of the Tg arm domains superimposed onto protomer A of the SARS-CoV-2 Spike protein (Wuhan strain; PDB ID: 6VXX (33)) using DALI superimposition results with MERS-CoV Spike protein protomer A as a reference (**Figure 6B**). Importantly, the regions on the Spike proteins that are found to be structurally similar to the arm domain of thyroglobulin, are reported as being immunogenically relevant (34). We next concentrated on sequence similarity analysis of all potentially immunogenic patches predicted on thyroglobulin whose length totaled 15 residues or more, after removing any gaps/discontinuities, and inter-monomer jumps (*i.e. conformational epitopes)*. Then, each of these 43 filtered sequences was individually subjected to a search across a database of 17481 known SARS-CoV-2 epitopes from the Immune Epitope Database (IEDB) (33). The results (34 hits) are presented in **Supplementary Table S5** (35).

**Figure 6:**
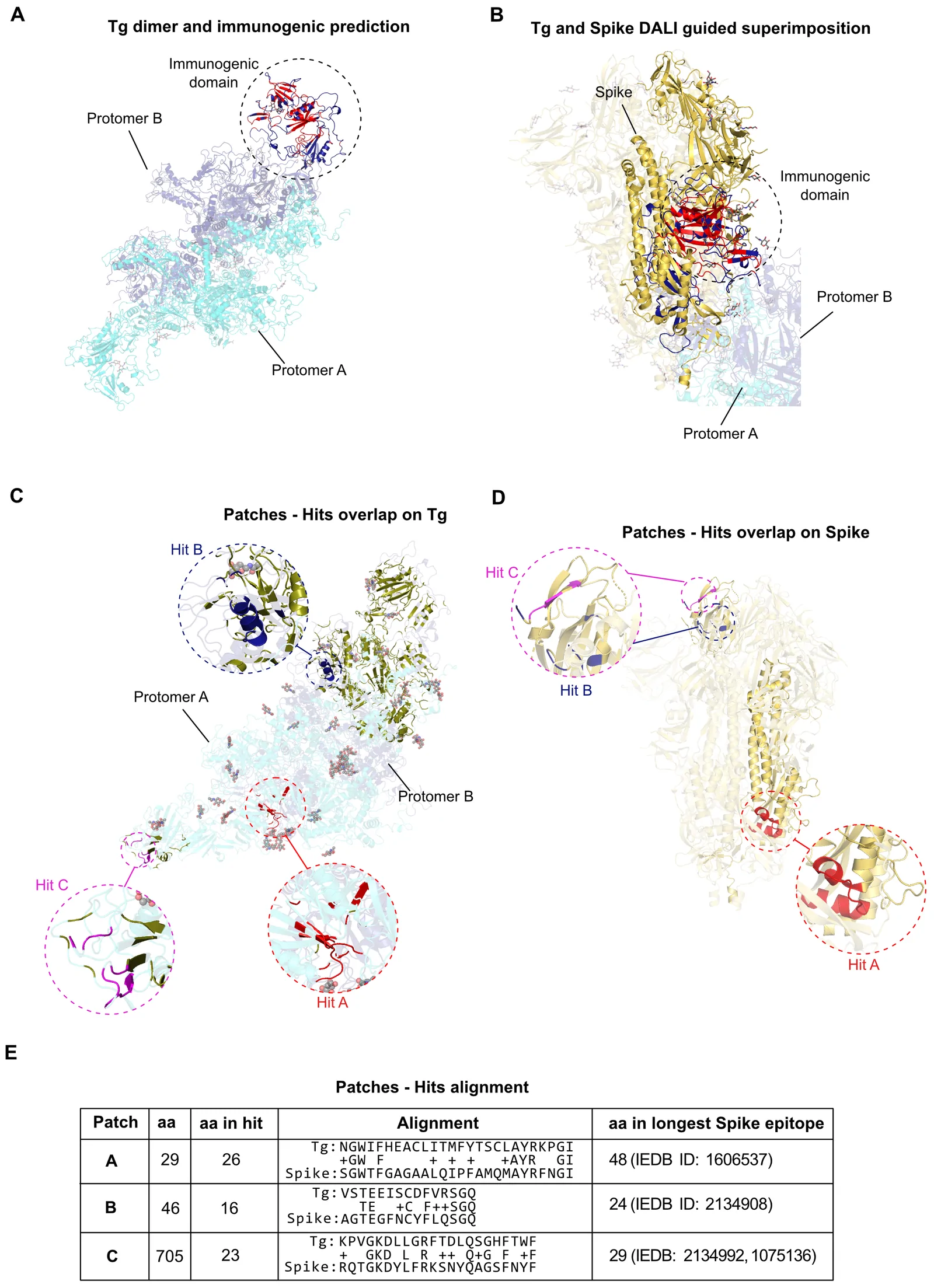
Similarity between SARS-CoV-2 Spike and thyroglobulin. **(A)** Thyroglobulin (Tg) dimer (protomer A: cyan; protomer B: blue), extracted from production frame 1 of our molecular dynamics simulation (see Computational Details), with the arm domain on protomer B (left) shown in full opacity and its predicted immunogenic regions highlighted in red. **(B)** DALI structural similarity search results: superimposition of arm domain onto protomer A of the SARS-CoV-2 Spike protein (yellow; protomer A in full opacity; PDB ID: 6VXX) using MERS-CoV Spike protomer A as the reference. **(C)** Location of patches A, B, and C (see main text; olive) on the thyroglobulin dimer, and locations of hits A, B, and C within these patches (red, magenta, blue, respectively). The three patch A residues not comprised in hit A are barely visible, and hence unlabeled. Other residues are rendered transparently (protomer A: cyan; protomer B: blue). Heavy atoms of patch C glycans are shown as olive spheres; remaining glycans: N: blue, C: grey, O; red. The structure comes from frame 1 of our molecular dynamics simulation (see Computational Details). **(D)** Location of known epitope sub-portions on protomer A of the SARS-CoV-2 Spike protein (PDB ID: 6VXX) that are sequentially similar to hits A–C (per BLASTp). Protomer A in full opacity; B and C more transparent. **(E)** Aminoacidic (aa) sequence alignment of hits from patches A, B, and C found on thyroglobulin (see C, D, and **Supplementary Table S5**) with portions of known SARS-CoV-2 Spike proteins, as output by BLASTp. Glycan heavy atoms are rendered as sticks in panels A to C (red: O, grey: C, blue: N).

In particular, one linear epitope localized in the S2 domain and two potential conformational epitopes found in the receptor binding domain (RBD) (**Figure 6B**) were found on the thyroglobulin dimer (**Figures 6C, D**) and emerged as ‘best hits’ also as sequential mimicry (**Figure 6E**).

## 4 Discussion

Many viral infections have been known to affect the endocrine system (36), such as human immunodeficiency virus type-1 (HIV-1) (37), coxsackieviruses B (38), and SARS-CoV-2 (39).

Due to the high incidence of infections and reinfections with SARS-CoV-2, it is very important to understand how this viral infection can potentially cause thyroid damage.

COVID-19 has been associated with different thyroid dysfunctions (40), which can have important health implications. Both new onset and the recurrence of autoimmune thyroid conditions, such as Graves’ disease and Hashimoto’s thyroiditis, have been reported after COVID-19, raising concerns about the potential role of the virus in triggering autoimmunity (41, 42). Moreover, as described in other viral infections (6), SARS-CoV-2 may cause thyroid inflammation and damage with temporary thyroid dysfunction, irrespective of preexisting thyroid disorders, due to the occurrence of subacute thyroiditis (43, 44). Furthermore, COVID-19 can induce a perturbation of hypothalamus-pituitary-thyroid axis and the occurrence of NTIS, especially in severe cases characterized by critical general conditions (7, 8). In case of severe COVID-19, a form of silent painless thyroiditis associated with NTIS has also been described, resulting from the likely combination of NTIS with subacute thyroiditis (3, 45).

In part, these disorders are considered as secondary to the local virus infection and replication within the thyroid gland cells. Like other endocrine tissues, the virus can attack the thyroid gland using ACE2 receptors which have a high expression level in thyroid cells (9, 11), and can potentially disrupt hormone production and regulation. Indeed, SARS-CoV-2 genome and proteins have been found within thyroid cells of patients affected with COVID-19 (46, 47). The direct effect on the thyroid gland is due to the cytotoxic effect of the virus on the target cell, while the indirect effect is caused by the aberrant immune inflammatory responses. Concerning the immune system, it is known that both innate and adaptive immune responses are regulated by thyroid hormones, including cytokine production and release (48). On the other hand, systemic viral infections, including SARS-CoV-2, may result in a “cytokine storm” that also affects the hypothalamus-pituitary-thyroid axis and thyroid function (49). However, the immunological mechanisms specifically contributing to TD in severe forms of COVID-19 are not fully understood.

Multiple studies have highlighted the association, induction, and possible roles of various types of autoantibodies in SARS-CoV-2 infection. Indeed, the primary categories are anti-ACE2, anti-cytokine antibodies (ACAs), antinuclear antibodies (ANAs), anti-phospholipid antibodies (APLAs), and anti-rheumatoid factors (RFs) (50).

Our protein array study highlighted a remarkable IgG autoreactivity of individuals who were recovering from SARS-CoV-2 infection and led to the identification of 358 recombinant human proteins that were specifically recognized by them, compared to healthy individuals. Among these, 17 were associated with the plasma membrane or secreted as signaling molecules. Some of these proteins were already reported as autoantigens such as IFNγ (51–53), while most of them were not previously reported. With the aim to identify autoreactive antibodies with a potential immuno-pathogenic role during COVID-19, we focused on CD16A (FcγRIIIa) and KIR2DL1 which also displayed a high immunoreactivity in individuals who developed or showed enhancement of TD during the acute phase of the infection. KIR2DL1 is a member of the Killer cell immunoglobulin-like receptors (KIRs), transmembrane glycoproteins expressed by natural killer (NK) cells and subsets of T cells, with a long cytoplasmic domain that transduces inhibitory signals upon ligand binding via an immune tyrosine-based inhibitory motif (ITIM). KIR2DL1 down-regulates the cytotoxicity of NK cells upon recognition of specific class I major histocompatibility complex (MHC) molecules on target cells (54).

Concerning CD16A, it is highly expressed on the surface of NK cells, neutrophils, monocytes, macrophages, and is an activating receptor for the immunoglobulin Fc portion involved in NK cell-mediated antibody-dependent cellular cytotoxicity (ADCC) responses. During SARS-CoV-2 infection, NK cells recognize IgG opsonizing SARS-CoV-2-infected cells and cell-free virions via CD16A, leading subsequently to the release of proinflammatory cytokines, such as IFNγ and TNFα, as well as of cytolytic mediators, such as perforin and granzyme B, which cause ADCC (55).

Overall, NK cells play a controversial role in SARS-CoV-2 infections, as it is unclear whether proinflammatory and cytotoxic SARS-CoV-2-specific responses limit disease severity or rather contribute to the immunopathogenesis of severe COVID-19 (55, 56). In line with ELISA data, we found that sera of patients with TD in the context of severe COVID-19 promoted activation of NK cells, being able to bind to and induce degranulation more efficiently than sera of individuals with Graves’ disease and healthy controls, indicating an immunomodulating activity. It is known that the binding ability of IgGs to CD16A is modulated by the fucosylation state of IgGs, since afucosylated IgGs have an enhanced binding up to ∼40 fold to CD16A in comparison to their fucosylated counterparts. This results in increased cytokine production and cellular cytotoxic responses.

In healthy conditions, the majority of IgGs found in plasma are fucosylated (∼94%) (57). However, afucosylated antigen-specific IgG responses have been described in various infectious diseases, including those caused by HIV (58), *Plasmodium falciparum*, secondary Dengue infections (59) and SARS-CoV-2 (27, 60). Notably, SARS-CoV-2-specific afucosylated IgGs were found in critically ill COVID-19 patients but not in individuals with mild symptoms. Over the 2 weeks after seroconversion, the amount of fucosylated anti-SARS-CoV-2 IgGs increased markedly, in most patients reaching relative levels similar to those found in total IgGs (27). Afucosylated IgG immune complexes isolated from COVID-19 patients induced inflammatory cytokine production and robust infiltration of the lung by immune cells (61). In our study, we found that polyclonal anti-Spike IgGs of patients with TD while affected by severe forms of COVID-19 (3, 4), showed a lower fucosylation state than those from individuals without any thyroid disease. Such an effect appeared to be a peculiarity of anti-Spike antibodies, as it was not observed on total serum IgGs. We hypothesize that such afucosylation levels correlate with low expression of fucosyltransferases in spike-specific plasma cells (62), such as FUT8 (α1, 6-fucosyltransferase) that catalyzed N-glycan core fucosylation at position 297.

Moreover, we observed that sera from individuals with COVID-19, including those with TD, were able to bind more efficiently to immortalized human thyroid epithelial cells than sera from healthy donors. We also observed that the presence of recombinant Spike in the assay promoted a higher binding to thyrocytes than without Spike, a feature even more evident in COVID-19-TD samples. Indeed, human thyroid epithelial cells physiologically express ACE2 (10, 63) that, during infection, interact with SARS-CoV-2 viral particles through the Spike protein. This event can facilitate an accumulation of anti-Spike IgG antibodies bound to and/or in close proximity to the thyroid epithelial tissue.

Collectively, our findings support the hypothesis that highly autoreactive antibodies elicited during SARS-CoV-2 infection may contribute to the increased binding to immortalized human thyroid epithelial cells, with potential inflammatory consequences. Although further studies are needed to assess the connection among our observations, the presence of anti-Spike antibodies, with low fucosylation level, and activated NK cells could further worsen the local inflammation. Since NK degranulation is considered as a highly sensitive and reliable surrogate for antibody-dependent cell cytotoxicity (ADCC) (64, 65), we hypothesize that a local accumulation of afucosylated IgG1 antibodies bound on thyroid cells, could engage NK cells and trigger the cytotoxic machinery in the thyroid tissue. However, future studies using patient-derived monoclonal antibodies isolated during acute infection and real-time killing assays on standardized thyroid cell lines or organoids will be required to precisely assess the mechanism and quantify the rate of cell death.

Moreover, we hypothesize that the increased binding of serum antibodies to immortalized human thyroid epithelial cells could implicate molecular mimicry, meaning the recognition of self-epitopes as external antigens because of a similarity between the autoreactive proteins and SARS-CoV-2 proteins. Historically, molecular mimicry is considered one of the potential mechanisms by which an infection may influence the development of autoimmune diseases, including thyroid autoimmune diseases (50). Several studies demonstrated the homology of several peptide sequences between human beings and SARS-CoV-2 proteins. Indeed, recent computational analyses of structural homology between SARS-CoV-2 antigens and the human proteome have identified several shared epitopes (66) also referred to as “molecular mimicry hot-spots” (67–69) with little overlap with our candidates, highlighting that multiple approaches are necessary to provide a broader picture of the interlink between autoreactive response and molecular mimicry events during SARS-CoV-2 infection. Interestingly, immunization of mice with the receptor-binding domain (RBD) protein, as demonstrated by Lai et al., generated antibodies capable of binding to ACE2, suggesting the presence of cross-reactive epitopes (70). Furthermore, SARS-CoV-2 Spike protein has been found to have a local 70% homology with thyroid peroxidase (TPO), one of the major thyroid autoantigens, and anti-Spike autoantibodies cross-react with TPO (71).

In our study, by computational analysis we found structural and sequence similarity between Spike and predicted immunogenic regions on thyroglobulin (Tg), a secreted protein highly enriched in thyroid glands and a typical autoantigen in patients with autoimmune TD. The similarity between SARS-CoV-2 Spike and thyroglobulin was also reported by Churilov et al. (72), further strengthening the possibility of an autoreactive response due to a molecular mimicry between these proteins. By using a different approach (*i.e.*, a systematic scan of the entire thyroglobulin sequence in five-residue intervals), these authors found three pentapeptidic sequences in thyroglobulin that matched three SARS-CoV-2 Spike epitopes present in the IEDB. All three peptide sequences were captured either in part or in full by our MLCE conformational epitope predictions. These data provide preliminary observations that may inform the hypothesis of structural similarity between viral and host proteins, which will require further experimental investigation.

The primary limitation of this study is the relatively small number of patient samples taken during hospitalization for COVID-19, with paired thyroid assessment and stored sera available for subsequent characterization of the antibody response. This is because laboratory tests for thyroid dysfunction were routinely included at hospital admittance only in few hospital wards, and the collection of additional sera during hospitalization for subsequent analyses took several months to be organized, due to pandemic restrictions. Furthermore, COVID-19-related transient AT affects only 10-15% of patients with severe COVID-19 (3, 4), thus further reducing the number of COVID-19 patients with both TD and available stored sera for characterization of the antibody response. Due to such limitations, we could not perform sub-analyses further stratified by TD. Among other variables, correlation analysis with patients’ HLA-type did not give meaningful results (not shown in the manuscript).

Considering the necessity to identify a control cohort of patients with thyroid dysfunctions and available stored sera from the pre-pandemic era, it was not possible to apply corrections for age and sex. Thyroid disorders affect women from 4 to 12 times more frequently than men, depending on the specific disease (13). Such asymmetric sexual distribution explains why Graves’ disease patients in this study were predominantly females, in contrast to COVID-19-related-TD patients who were predominantly males, reflecting the known role of male sex as a risk factor for severe COVID-19 (73). Despite this limitation, sex should not represent a significant confounding factor in this study, since Graves’ disease patients showed similar results to healthy controls, who were age- and sex-matched with COVID-19-related-TD patients.

A second limitation is that the study design did not allow longitudinal sampling from the same patients, thus preventing an evaluation of the duration of the autoreactive response and of the low fucosylation of anti-Spike antibodies. It has been well-documented that afucosylated anti-Spike IgGs are a hallmark of severe acute COVID-19 that typically normalize as the viral antigen is cleared and the immune response matures. Our study represents a snapshot of the acute pathogenic responses that typically evolve toward memory B-cell formation and glycan normalization (74). Finally, the lower fucosylation state of anti-Spike antibodies raises questions on the molecular and genetic explanations that warrant future investigation. Based on other reports (75, 76), we assume that such state could be ascribed to a defective FUT8 expression in antigen-specific B cell subsets. However, due to the emergency situation, PBMC samples of the patients who developed TD in the context of severe COVID-19 were not routinely stored during the hospitalization phase.

Another limitation of the study is that the relatively low quantity of sera prevented the purification of sufficient amount of specific anti-Spike IgGs for different functional assays. For this reason, we could not expand the analysis of NK activation. Similarly, we could not demonstrate that specific anti-Spike IgGs were directly responsible for the killing of immortalized human thyroid epithelial cells via an Fc-mediated mechanism. This is also because only human IgG1, a subset of the entire polyclonal population of Spike-specific antibodies, could contribute to Fc-mediated activity. In addition, the NK-cell activation assays was performed using whole serum samples, which allows to assess the potential effect of afucosylated anti-Spike antibody in more physiologic conditions. However, this setting did not allow to determine whether the observed NK-cell activation was exclusively attributable to the afucosylation of anti-Spike IgG or was also influenced by their higher abundance in the serum of COVID-19-TD. Although relative Fc fucosylation was precisely normalized for ELISA profiling, we cannot completely rule out total anti-Spike IgG concentration as a potential confounding variable.

Despite these limitations, our study offers valuable immunological insights into the altered antibody response to SARS-CoV-2 infection in patients with TD during the acute phase of the infection resulting in severe COVID-19.

## Data Availability

The original contributions presented in the study are included in the article/**Supplementary Material**. Further inquiries can be directed to the corresponding authors.
