# Supplementary material for: Antibody afucosylation and enhanced autoreactive antibody response are associated with thyroid dysfunction during severe SARS-CoV-2 infection
Source: Front Immunol. 2026 Oct 19;17:1806272. doi: 10.3389/fimmu.2026.1806272 (PMC13724446; doi:10.3389/fimmu.2026.1806272)
Supplement: Supplementary file 1 [file DataSheet1.docx]

Supplementary Material

# 1 Supplementary Figures

**Supplementary Figure 1. Sera from patients with COVID-19-TD show higher amounts of anti-SARS-CoV-2-S antibodies.** Data are expressed as the signal of the analyzed sera normalized on the internal control (S/Co). The average positive signal value was approximately 8.3 for the COVID-19 (n = 17) and 11.7 for the COVID-19-TD group (n = 11).

**Supplementary Figure 2. Sera of the COVID-19-TD cohort are able to specifically bind CD16A present on the membrane of NK cells.** (**A**) Flow cytometry analysis of NK cells using commercial anti-CD16A antibody pre-incubated with increasing concentrations of recombinant CD16A (range 0 µg/ml – 25 µg/ml). The curve shows the residual binding of the antibody to endogenous CD16A present on the cell membrane, following competition with the recombinant protein. The values shown are expressed as a percentage of the cells that have bound the antibody, using secondary anti-human-IgG/APC antibody for detection. (**B**) Flow cytometry analysis of NK cells using sera from the HC (black lines, n = 4) or COVID-19-TD groups (red lines, n = 5) pre-incubated with increasing concentrations of recombinant CD16A (range 0 µg/ml – 25 µg/ml). The curves report the IgG binding to endogenous CD16A present on the NK cell membrane following competition with the recombinant protein. The values shown are expressed as a percentage of the positive cells detected by the secondary anti-human-IgG/APC antibody. (**C**) and (**D**) Flow cytometry analysis of NK cells using HC (circle) or TD (triangle) groups sera. IgG binding is detected by incubation with the secondary anti-human-IgG/APC antibody and is represented as the percentage of positive cells in panel C or Relative Fluorescence Intensity (RFI) in panel D. The dotted line represents the positivity threshold calculated on the average of HC + 1 standard deviation. Statistical analyses were performed using Mann-Whitney t test. P value: * < 0,05.

**Supplementary Figure 3*.* Gating strategy to identify CD16A^+^CD56^dim^ NK subset expressing CD107a.** Flow-cytometry gating strategy. Gating hierarchy of a representative sample (NK cells from healthy control). From left to right and from up to down: (a) in FSC-A versus SSC-A, identification of the cloud of cell-like events; (b) in FSC-A versus Live and Dead, identification of live cells; (c) FSC-A versus CD3, identification of CD3 negative cells; (d) FSC-A versus CD16A, identification of CD16A positive cells (among CD3 negative cells; (e) FSC-A versus CD56, identification of CD16A^+^CD56^dim^ subset of NK cells characterized by high cytotoxic activity; (f) identification of CD16A^+^CD56^dim^ NK cells expressing CD107a.

**Supplementary Figure S4**. The binding to CI-huThyrEC cells is increased by prior incubation with recombinant Spike. CI-huThyrEC cells (200,000 cells/well) were incubated with/out recombinant Spike (1 µg/ml) and further incubated with sera of COVID-19 group 2 and COVID-19-TD with well detectable anti-Spike antibody (titer > 3200). IgG that bound to the thyrocyte plasma membrane was detected by anti-human IgG/Alexa Fluor 647 antibody and analyzed by flow cytometry. The binding increase in the presence of Spike is reported as fold increase compared to cells not incubated with Spike. Statistical analyses were performed using one-way ANOVA test. P value: * < 0.05.

**Supplementary Figure 5. Lectin blot of AAL for Spike-specific IgG derived from TD sera indicates a reduction in the FC core fucosylation.** Detection of core fucosylation by lectin blot. **A)** HRP-conjugated AAL (0.6 μg/mL) was used for lectin blotting with different glycosylated and non-glycosylated IgG: 0,5 ug SARS-CoV-2-Spike IgG derived from TD sera (lane 1); 3 μg of afucosylated mAb (mAb Afuc) (lane 2); 3 μg of fucosylated mAb (mAb Fuc) (lane 3). After incubation with the lectin, the membrane was visualized using chemiluminescent substrate. **B)** Quantification of IgG loaded per lane. The membrane was incubated with an anti-human IgG conjugated with HRP and the targets were visualised using a chemiluminescent substrate. Band quantification using Fiji was used to normalise the effective ratio with the AAL. Densitometric analysis was used to compare the normalized intensity of the lectin blot bands and was expressed as a percentage of fucosylation. Hc: heavy chain; Lc: light chain.

**Supplementary Figure 6. The level of fucosylation of total IgG derived from COVID-19-TD sera is comparable to that of healthy controls.** IgG-Fc core fucosylation levels of total IgG in COVID-19-TD sera and HC, measured by an ELISA-lectin assay. Total IgG were purified for each serum and quantified. For each individual sera, 1 μg of eluted IgG was used. Measured OD values (fucosylation) correspond to the binding of HRP-conjugated AAL lectin to IgG fucose. The fucosylated mAb (mAb Fuc) antibody, which was employed under the same conditions, was used as reference for 100% fucosylation. Each dot represents one individual sera.

**Supplementary Figure 7. Specific recognition of CD16a by patients sera.** Recombinant CD16a was coated onto the plate and pre-incubated with a commercial afucosylated mAb (Biosynth, CLA1499) at a saturating concentration (20 μg/mL), followed by incubation with a pool of COVID-19 sera. Compared with wells lacking mAb pre-incubation, the increased OD450 signal observed under these conditions demonstrated that the interaction with CD16a is driven specifically by an IgG Fab mediated binding mechanism.

# 2 Supplementary Methods

**2.1 Human Protein array data analysis**

*Image processing, intra- and interarray variability.* The data processing includes a range of sequential statistical functions organized into an R package. In HuProt arrays version 4.0 proteins are printed in duplicate on a slide and controls proteins spotted in each subarray divided into blocks. For each sample, the raw median fluorescence intensity (MFI) values of each spot were measured, signal-to-local-background ratios were calculated using ImaGene 8.0, and spot morphology and deviation from the expected spot position were considered using the default ImaGene settings. The background is usually calculated using a circular region around the spot. For each sample, the background-subtracted MFI of replicated spots was determined and a Coefficient of Variation (CV) was calculated on replicate spots (for Intra- and Interarray variability) and help to quantify the reliability of the experimental procedures. Each protein was checked for displaying a CV correlated to its MFI. If the CV value, defined as CV= σ/μ where σ is the standard deviation and μ is the mean of the signal at original scale, was not within the expected range the protein was not considered for further analysis.

*Normalization methods and proteins selection.* To minimizes the mean–variance dependence (MVD) data exported were normalized by robust linear model normalization (RLM) for microarray data. This method stabilizes the mean–variance dependence (MVD) by using standardized control spots on each slide to adjust intensities across the entire experiment (1). In brief, methods assumes that the signal detected from control spots remains constant with the exception of technical differences within or between slides. It is the method of choice when a significant amount of variation between samples is anticipated. Data from control spots are fitted to a robust statistical model using an iteratively reweighted least-squares procedure with a robust ‘‘sandwich estimator”, like the median. Fixed effects for each array or slide and positive control proteins are estimated from the statistical model. After Log_2_ transformation, the geometric average MFI for each protein was calculated using samples from healthy subjects known to be uninfected with SARS-CoV-2 (all obtained before 2019).

*Differential expression analyses.* Differentially reactive proteins between the experimental groups, HC, during and post-infection, were identified with the MFI equal to 10000, and filtered on the basis of adjusted P value (<0.05) and log2 fold change (≥ 2).

**2.2 CI-huThyrEC**

CI-huThyrEC are Immortalized Human Thyroid Epithelial Cells, which are also known as follicular cells or thyrocytes. These cells were purchased from InSCREENex (INS-CI-1017) coated with Epithelial Cell Coating Solution, washed with phosphate-buffered saline (PBS 1x) and cultured in the media provided by the manufacturer (INS-ME-1017, InSCREENex). The cells were maintained in culture and detached from flasks using Accutase (Thermo Fisher Scientific). After that, cells were washed in PBS 1× stained in 96 round bottom plate as 200.000/well and added a serum (1:50) for 40 min at RT. When indicated, cells were incubated with/out recombinant Spike (1 ug/ml) for 30 minute 37 °C, before serum addition. After washing in MACS buffer, cells were stained with goat anti-hu IgG Alexa fluor 647 Invitrogen 1:200 for 30 min at 4 °C. All incubation steps were performed in the dark. The experiment was read on FACSCanto I.

**2.3 Structural mimicry investigation**

To investigate mechanisms of structural mimicry among potential antigens, we first set out to identify all thyroid proteins with homology to the SARS-CoV-2 Spike protein by running a MAFFT search (2). Construction of a complete glycosylated thyroglobulin dimer was begun from the (incomplete) CryoEM structures with PDB IDs 7B75 and 6SCJ (3,4) as the initial references. Missing loops (Supplementary Table 6) and bisulfide bridge (Table S7) were added and modelled as fully described in the supplementary information. The constructed dimer was then subjected to a short round of molecular dynamics (MD) simulation. Potential epitopes were predicted by applying the Matrix of Lowest Coupling Energy method (MLCE) (5–10) (<https://github.com/colombolab>) to the resulting MD trajectory. A structural similarity search of the largest epitope predicted on thyroglobulin, isolated from frame #1 of its MD trajectory, was conducted online across all structures released in the PDB at the time of writing, on the DALI server (11). In order to identify any viral proteins present in the search results, these were filtered using the case-insensitive keywords “Spike”, “MPro”, “virus” and “viral”. Sequence similarity searches for predicted thyroglobulin conformational epitopes were conducted across the 17481 known SARS-CoV-2 epitopes released in the Immune Epitope Database (IEDB; (12)) at the time of writing. The search itself was conducted locally, using the BLASTp tool (version 2.16.0+) downloaded (13) as part of the BLAST+ platform (14). This required downloading the SARS-CoV-2 epitope sequences from the IEDB and converting them to the fasta format using an in-house script. BLASTp was run with default settings.

**2.4 Immunological Insight on Structurally Similar Regions between Thyroglobulin and Spike Protein**

An in-dept analysis of DALI results (11) (main text, Fig. 7B) reveals immunogenically relevant regions on the Spike protein (15) that overlap with the arm domain of thyroglobulin. Indeed, the domain overlaps with the furin cleavage site (SARS-CoV-2 residues 680–685) (16). Additional areas of structural overlap are found close to the N-terminal domain residues V36 and Y38, which are involved (15), respectively, in interactions with Toll-like Receptor 1 and Toll-like Receptor 6; overlap with another potential (15) immunogenic site (only identified computationally as possibly recognized by Toll-like Receptor 4) is with N657 in the S1 domain. Finally, the arm domain is also seen to overlap well with the S2 domain of the SARS-CoV-2 Spike protein: in this region, structural overlap stands out because residues 936-952 (in heptad-repeat domain 1) and residues 1011-1028 are all involved in T-cell receptor recognition (15).

**2.5 Full Results from the IEDB Sequence Similarity Analysis**

The 34 hits resulting from our similarity search of the 43 conformational epitopes chosen amongst those predicted by MLCE are presented in Table S5, sorted from the best (lowest) to the worst (highest) expectation value (E value); individual hits are also listed with their corresponding Bit-score (17). Of course, hits do not comprise queried patches in their entirety, some of which were considerably long: typically, a hit corresponds to a subportion of a patch that is found to have a high sequence similarity with a subportion of a known epitope.

Since it is reported by Pearson (17) that acceptable E values for proteins tend to fall below 0.001 and acceptable bit-scores tend to exceed 40-50 bits, one can see that none of the queried immunogenic patches in Supplementary Table 5 stand out for being having a particularly high degree of homology to known SARS-CoV-2 epitopes. That said, there emerge three parent patches with hits having E values below 1. While this is still 3 orders of magnitude over the recommended E value threshold, it is certainly plausible to view these patches as potential starting points for future experimental cross-reactivity validation. Despite these three patches ‘occupying’ 7 entries of the thyroglobulin half of Table S5, we confirm that each hit represents an instance of the same subportion of each parent patch being recognized as similar to an identical subportion of similar SARS-CoV-2 epitopes (in essence, seven different entries, but representing three unique patch-epitope matches). This is possible because several IEDB entries are redundant or semiredundant, with differently sized epitopes sharing a common residue core. Incidentally all three matching epitopes are located on the Spike protein.

These are the hits that we present in the main text (Fig. 7E). More specifically, the first of these hits (henceforth hit A) is contained within the 29-residue patch number 27 (henceforth patch A) coming from the MLCE search conducted with a 10% cutoff. The second hit (B) is contained within the 46-residue patch number 16 (patch B) emerging from the MLCE search with a 5% cutoff. The third hit (C) is located within the vast 705-residue patch number 15 (patch C), found by the MLCE search conducted with a 15% cutoff.

**2.6 Construction of a Thyroglobulin Model**

Thyroglobulin (3) was very complex to model. It consists of two protomers, each of which has 2768 residues, 7 glycosylation sites, and at least 58 intra-protomer disulfide bridges. Furthermore, the two available CryoEM structures—PDB IDs: 7B75 (4) and 6SCJ (3) —both feature large unresolved portions, and don’t overlap in some of the disordered regions. For all these reasons, preparation of thyroglobulin for simulation was approached differently, with the MODELLER package (18) (version 10.5) to model and refine missing regions.

Protomer A of 7B75 served as our main template for model construction, in light of its better resolution (3.2 Å *vs.* 3.6 Å); however, since 6SCJ has some resolved areas that are not present in 7B75—including one that comprises a disulfide bridge—some portions of our model were derived from 6SCJ if this was found to be structurally feasible. Once all regions of protomer A were modeled, we used *PyMOL* (The PyMOL Molecular Graphics System, Version 3.0 Schrödinger, LLC.) to stitch identical copies into protomer B.

We note that all iodotyrosines were modeled as normal tyrosines for simplicity, and that protomer A of 7B75 also served as the sole template to model glycosylation, since more glycans were resolved compared to 6SCJ. We did not model additional glycans.

**2.7 Missing Regions.** The 12 missing regions that we modeled (*N-* and *C-*termini; 10 loops) are listed in full in Table S6; modeling was carried out using the MODELLER suite (18) (version 10.5) parallelized on 10 CPUs. As stated in the main text, protomer A of CryoEM structure with PDB ID 7B75 (4) as the main template; however (Table S6), *N-* and *C-*termini and loop 10 also incorporated some (extra) residues that were resolved in 6SCJ (3) only. Adjacent residues were sometimes remodeled together with the missing regions. The full thyroglobulin sequence downloaded from UniProtKB (<https://www.uniprot.org/>) served as a reference to determine amino acid sequences of the parts missing from the reference CryoEM structures. Each missing region was modeled separately. The next few paragraphs provide an overview of the procedure. First of all, MODELLER was instructed to generate 10 models of the loop or terminus to be modeled, using the DOPE-High-resolution (DOPE-HR) statistical potential (19). The predefined “slow” optimization procedure (*i.e.*, the most thorough one of several available) was then applied to initially refine each generated model of each missing loop/terminus through repeated rounds of conjugate gradient minimization over increasingly large residue ranges. Each time it was called, the conjugate gradient algorithm was allowed to run for up to 300 iterations. After repeated minimization, each model was then refined by means of a “slow” MD annealing procedure (*i.e.*, the third slowest of several available). First, this entailed slowly heating each model from 0 K to 1000 K over a total of 1100 steps with a 4 fs time-step. There followed cooling from 1000 K back to 300 K over an even longer period (4800 steps). Once minimization and MD annealing were run for a first cycle, MODELLER was always instructed to repeat them in full for a second cycle (*i.e.*, by reinitiating the “slow” optimization procedure on loop/terminus models just issued from the MD annealing). Out of the 10 models that were generated for each of the two termini and each of the 10 missing loops (Supplementary Table 6), the model to ultimately insert into 7B75 was picked after ranking all 10 models by three different MODELLER scores (19)—DOPE-HR, DOPE, and molpdf (molecular probability density function)—and choosing the one that most consistently scored best, provided it was structurally plausible upon visual inspection. Since loops were modeled in the absence of protomer B, we also ensured that loops touching the inter-protomer interface did not result in steric clashes once protomer B was reintroduced in the structure.

**2.8 Disulfide Bridges.** In each protomer, disulfide bridges were modeled between the following 58 pairs of cysteines, shown in Table S7.

**2.9 Preparation of the Thyroglobulin Monomer for Simulation**

- **General Strategy.** After preparing the thyroglobulin model as discussed above, the *reduce* utility (20) distributed with AmberTools (21,22), the *PropKa* pH prediction tool, and the AmberTool *tleap (21,22)* were applied in succession. Tasks performed by these operations include: (1) verification of the preferred orientations of histidine, non-glycosylated asparagine and glutamine sidechains; (2) assessment of histidine tautomerization states; (3) addition of different classes of protons (including on some protonated titratable residues at pH 7 found on thyroglobulin and on its glycans); (4) addition of suitable capping groups at *N-* and *C-*termini of each protomer (–NH_3_^+^ and COO^–^ groups, respectively); and (5) formalization of disulfide bridges and Asn-glycan and glycan-glycan bonds. Steps (4) and (5) are carried out with the AmberTool *tleap (21,22)*. Thereafter, again with *tleap*, the system is solvated in a cuboidal box of water (TIP3P model (23)), and ions were added (24) to recreate a neutral system with a 0.1 M ionic strength. Finally, there followed a round of preminimization with AmberTool *parmed* (21,22) to relax the modeled loops and any additional clashes arising at the inter-protomer interface.
- **Details on Tautomerization and Protonation States.** Going into more detail about tautomers and protonation states, 28 of the 39 histidines on each thyroglobulin protomer are predicted to protonated on their Nε2 atom, whereas histidines 236, 892, 1295, 1384, 1429, 1669, 1977, 1882, 2486, 2568, and 2661 are predicted to be protonated on their Nδ1 atom. Assessment of protonation states of titratable residues (including histidines) at pH 7 predicted aspartates 239, 831, and 2586 on each protomer to be protonated, as well as glutamates 244, 251, 811, and 1420 on each protomer.
- **Details on Solvation.** In building the cuboidal box of TIP3P water (23), we ensured that every solute atom was at least 12 Å from the closest box edge. The ionic force of 0.1 M was recreated in the simulation box using the method recommended by Machado and Pantano (24), resulting in 509 Na^+^ + 417 Cl^–^ ions (charge of –92).

**2.10 Forcefield and Cutoff Parameters Used in our Simulation**

Water molecules were, as stated, modeled according to TIP3P parameters (23), while ions were treated with the parameters developed by Joung and Cheatham (25). All standard protein residues were treated using the *ff14SB* forcefield (26). For glycans and *N-*glycosylated asparagines, we adopted the *GLYCAM06j* forcefield instead (27). Throughout our MD simulations, we imposed a cutoff of 8 Å for the evaluation in direct space of Lennard-Jones and Coulomb interactions that each atom makes with its nonbonded partners. Beyond this, only Coulomb interactions are calculated, in reciprocal space, using the Particle Mesh Ewald approach (28).

**2.11 Molecular Dynamics Simulation**

After preparing, protonating, solvating, and neutralizing the thyroglobulin model as discussed, we ran a single Molecular Dynamics (MD) simulation lasting only 1 ns (production stage). This was run with the *AMBER* molecular simulation suite (21). Preceding the production stage were: (1) 600 minimization steps (300 on non-water Hs only, 300 on all atoms); and (2) a 4.069 ns MD preproduction stage comprising solvent equilibration, heating, and solvent-solute equilibration with increasingly lax restraints on the thyroglobulin backbone. We list these in more detail over the next subsections.

*Minimization***.** MD preproduction begins with two successive rounds of structural minimization, both carried out with the *sander* tool (21,22). Each round consists of 300 steps (10 with the steepest descent algorithm and 290 with the conjugate gradient algorithm): the first round only involves solute hydrogens, while the second one involves all molecules and ions present in the system.

*Solvent equilibration***.** After minimization, there follows a round of solvent equilibration in the *NVT* ensemble, with temperatures in this case enforced by the Berendsen thermostat (29). Solvent equilibration is achieved by means of a rapid simulated annealing algorithm, which is applied over 9 ps to solvent molecules alone: all non-water molecules, including ions, are kept frozen in space (harmonic restraint constant, *k*: 10 kcal mol^–1^ Å^–2^ in the three Cartesian directions). The first 3 ps see the temperature rapidly rise from 25 K to 400 K; from picosecond 3 to picosecond 6, the temperature is maintained at 400 K; and for the final 3 ps, the solvent is cooled back to 25 K. Coupling to the heat bath τ is very tight for the first 6 ps, at 0.2 ps; at the start of cooling, it is immediately relaxed to 2.0 ps, and gradually retightened to 1.0 ps by the end of the cooling.

For this initial stage only, we introduce a shorter time-step (1 fs), so that solvent bonds containing hydrogen can be simulated unconstrainedly; subsequent stages are all run with a 2 fs time-step, which mandates the application of the *SHAKE* and *SETTLE* algorithms (30) to constrain hydrogen-containing bonds.

*Restrained heating of solute and heating of solvent.* After solvent equilibration, atomic velocities are randomly reassigned to all atoms so that they again reproduce an overall system temperature of 25 K; from this point on, simulation is continued using GPU-accelerated *pmemd.cuda (31)*. Retaining the *NVT* ensemble (but henceforth switching to the Langevin thermostat (32)), the system is gradually heated from 25 K to 300 K over 20 ps, with a rather weak coupling to the thermostat (collision frequency, γ: 0.75 ps^–1^): to prevent the protein in the system from unfolding, positional restraints are applied to all Cα atoms (*k*: 5 kcal mol^–1^ Å^–2^).

*Equilibration.* Equilibration follows heating and takes place over a total of 4.04 ns. Systems are immediately switched from the *NVT* to the *NpT* ensemble, imposing pressure stabilization at 1 atm (Berendsen barostat (29); coupling τ: 1.0 ps). Over the first 40 ps, restraints on Cα atoms are progressively released: for the first 20 ps, *k* is set to 3.75 kcal mol^–1^ Å^–2^; for the following 20 ps it is further lowered to 1.75 kcal mol^–1^ Å^–2^; and thereafter, all restraints are lifted. Similarly, coupling to the thermostat is progressively tightened: γ is set at 1.0 ps^–1^ for the first 2.04 ns, and 5.0 ps^–1^ for the final 2 ns. This ensures that for the final 2 ns of equilibration, conditions are identical to the MD production stage that immediately follows.

*Production.* The 1 ns production stage (2 fs time-step; 1 frame printed every 50 ps for a total of 20) is run with the GPU-accelerated *pmemd.cuda* utility (31) in the *NpT* ensemble (*T =* 300 K, enforced by the Langevin thermostat (32) with collision frequency 5.0 ps^–1^; *p =* 1 atm, enforced by the Berendsen barostat (29) with coupling constant 1.0 ps).

**2.12 The Matrix of Low Coupling Energies (MLCE) Method to predict potential epitopes**

The Matrix of Lowest Coupling Energy Method (MLCE) (5,6,33–36) was applied to all 20 frames resulting from the short MD simulation conducted for thyroglobulin, after stripping all ions and solvent molecules. Importantly, glycans are fully considered in the analysis and, if applicable, included in predicted epitopes. We briefly outline the analysis over the next few paragraphs, noting that it is entirely automated *via* our in-house script (<https://github.com/colombolab>).

*Preparatory stage: Minimization and MM/GBSA Calculations*. In the first stage, the protein conformation in each MD frame automatically undergoes 200 minimization steps in implicit solvent using the *AMBER* utility *sander* (21,22). Minimization of each stripped MD frame (10 steepest descent + 190 conjugate gradient) takes place in implicit solvent (generalized Born model; GB) (37). A cutoff of 12.0 Å is applied for the calculation of Lennard-Jones and Coulomb interactions in direct space; beyond this, only Coulomb interactions are calculated in reciprocal space using Particle Mesh Ewald (28). The implicit counterion concentration is 0.1 M at this stage. The solvent-accessible surface area is computed with the LCPO method (linear combinations of pairwise overlaps) (38). All of the above aspects are automatically taken care of by our in-house MLCE script (<https://github.com/colombolab>).

The MMPBSA.py script (39) in *AMBER tools* (21,22) is then invoked to carry out a MM/GBSA calculation (40) on each minimized structure (frame). The implicit solvation model used in MM/GBSA calculations is identical to the one used in the minimization stage that immediately precedes it, save for the implicit counterion concentration, which is set to 0.0 M, and the method to calculate the solvent-accessible surface area, which is switched to ICOSA (icosahedra are constructed around each atom, and progressively refined to become quasi-spheres).

*MLCE stage.* The total free energy estimated by MM/GBSA is conveniently broken down into individual contributions by every single residue-residue, glycan-glycan, or glycan-residue pair. For a protein of *N* residues + glycans, this will yield a symmetric *N × N* interaction matrix **M** whose elements *M_ij_* represent the interaction free energy between the *i*^th^ residue or glycan and the *j*^th^ residue or glycan.

**M** is diagonalized—*i.e.*, re-expressed as a product of eigenvalues λ_1_ to λ*_N_* and eigenvectors **ν**_1_ to **ν***_N_* such that

$$\mathbf{M}=\sum_{\alpha=1}^{N} \lambda_{\alpha}\boldsymbol{\nu}_{\alpha}$$

and λ_1_ to λ*_N_* are ranked from the most negative to the most positive.

The convenience of this diagonalization is that for compact, globular proteins, the first eigenvector **ν**_1_ ([ν_1_^1^, ν_1_^2^, …, ν_1_*^N^*]) associated with the most negative eigenvalue λ_1_ will only carry major contributions ν_1_*^i^* from residues/glycans *i* that are the most stabilizing energetically (ν_1_*^i^* components close or equal to 0 will come from residues that have no stabilizing function). For multidomain proteins such as thyroglobulin, Genoni *et al. (41)* showed that an additional number of essential eigenvectors are required on top of **ν**_1_ in order to best describe stabilizing interactions. These *N_e_* essential eigenvectors—selected automatically by our code—are the minimum number required to cover all residues/glycans in the protein with the minimum possible redundancy.

A simplified “essential folding matrix” $\tilde{\mathbf{M}}$ is thus constructed by our code as follows

$$\tilde{\mathbf{M}}=\sum_{\alpha=1}^{N_{e}} \lambda_{\alpha}\boldsymbol{\nu}_{\alpha}$$

only using these *N_e_* essential eigenvectors rather than all *N* of them. Filtering by spatially contiguous residue/glycan pairs, $\tilde{\mathbf{M}}$ is further simplified to ${\tilde{\mathbf{M}}}^{S}$, whose elements $\tilde{M}_{ij}^{S}$ are automatically set to 0 if the *i*^th^ and *j*^th^ residue or glycan are farther apart than an arbitrary 6 Å threshold, as measured from atom C_α_ or C_1_. Nonzero elements $\tilde{M}_{ij}^{S}$ with the *weakest* interactions—*i.e.* spatially contiguous residue or glycan pairs that are interacting but of minimal structural importance—are considered to be likely epitopes, ready for recognition by antibodies.

In this work, we run three separate rounds of MLCE analysis, examining epitopes derived from the weakest 5%, weakest 10%, or weakest 15% of interacting residue pairs. Amongst the differently sized epitopes predicted by our analysis, a few in particular were large enough to span specific protein domains almost in their entirety, particularly when MLCE epitope searches were conducted on the 10% weakest-interacting residue pairs. Indeed, the largest predicted epitope (*cf.* main text, Figure 7) came from this subset of epitopes.

**References**

1.	Sboner A, Karpikov A, Chen G, Smith M, Dawn M, Freeman-Cook L, Schweitzer B, Gerstein MB. Robust-linear-model normalization to reduce technical variability in functional protein microarrays. *J Proteome Res* (2009) 8:5451–5464. doi: 10.1021/PR900412K

2.	Katoh K, Rozewicki J, Yamada KD. MAFFT online service: multiple sequence alignment, interactive sequence choice and visualization. *Brief Bioinform* (2019) 20:1160–1166. doi: 10.1093/BIB/BBX108

3.	Coscia F, Taler-Verčič A, Chang VT, Sinn L, O’Reilly FJ, Izoré T, Renko M, Berger I, Rappsilber J, Turk D, et al. The structure of human thyroglobulin. *Nature* (2020) 578:627–630. doi: 10.1038/s41586-020-1995-4

4.	Adaixo R, Steiner EM, Righetto RD, Schmidt A, Stahlberg H, Taylor NMI. Cryo-EM structure of native human thyroglobulin. *Nat Commun* (2022) 13:61. doi: 10.1038/s41467-021-27693-8

5.	Lassaux P, Peri C, Ferrer-Navarro M, Gourlay LJ, Gori A, Conchillo-Solé O, Rinchai D, Lertmemongkolchai G, Longhi R, Daura X, et al. A Structure-Based Strategy for Epitope Discovery in Burkholderia pseudomallei OppA Antigen. *Structure* (2013) 21:167–175. doi: 10.1016/j.str.2012.10.005

6.	Morra G, Genoni A, Colombo G. Mechanisms of Differential Allosteric Modulation in Homologous Proteins: Insights from the Analysis of Internal Dynamics and Energetics of PDZ Domains. *J Chem Theory Comput* (2014) 10:5677–5689. doi: 10.1021/ct500326g

7.	Peri C, Gagni P, Combi F, Gori A, Chiari M, Longhi R, Cretich M, Colombo G. Rational Epitope Design for Protein Targeting. *ACS Chem Biol* (2013) 8:397–404. doi: 10.1021/cb300487u

8.	Marchetti F, Capelli R, Rizzato F, Laio A, Colombo G. The Subtle Trade-Off between Evolutionary and Energetic Constraints in Protein–Protein Interactions. *J Phys Chem Lett* (2019) 10:1489–1497. doi: 10.1021/acs.jpclett.9b00191

9.	Serapian SA, Marchetti F, Triveri A, Morra G, Meli M, Moroni E, Sautto GA, Rasola A, Colombo G. The Answer Lies in the Energy: How Simple Atomistic Molecular Dynamics Simulations May Hold the Key to Epitope Prediction on the Fully Glycosylated SARS-CoV-2 Spike Protein. *J Phys Chem Lett* (2020) 11:8084–8093. doi: 10.1021/acs.jpclett.0c02341

10.	Scantamburlo F, Masgras I, Ciscato F, Laquatra C, Frigerio F, Cinquini F, Pavoni S, Triveri A, Frasnetti E, Serapian SA, et al. Design and Test of Molecules that Interfere with the Recognition Mechanisms between the SARS-CoV-2 Spike Protein and Its Host Cell Receptors. *J Chem Inf Model* (2024) 64:8274–8282. doi: 10.1021/acs.jcim.4c01511

11.	Holm L. Dali server: structural unification of protein families. *Nucleic Acids Res* (2022) 50:W210–W215. doi: 10.1093/nar/gkac387

12.	Vita R, Mahajan S, Overton JA, Dhanda SK, Martini S, Cantrell JR, Wheeler DK, Sette A, Peters B. The Immune Epitope Database (IEDB): 2018 update. *Nucleic Acids Res* (2019) 47:D339–D343. doi: 10.1093/nar/gky1006

13.	Altschul S. Gapped BLAST and PSI-BLAST: a new generation of protein database search programs. *Nucleic Acids Res* (1997) 25:3389–3402. doi: 10.1093/nar/25.17.3389

14.	Camacho C, Coulouris G, Avagyan V, Ma N, Papadopoulos J, Bealer K, Madden TL. BLAST+: architecture and applications. *BMC Bioinformatics* (2009) 10:421. doi: 10.1186/1471-2105-10-421

15.	Magazine N, Zhang T, Bungwon AD, McGee MC, Wu Y, Veggiani G, Huang W. Immune Epitopes of SARS-CoV-2 Spike Protein and Considerations for Universal Vaccine Development. *Immunohorizons* (2024) 8:214–226. doi: 10.4049/immunohorizons.2400003

16.	Li L, Gao M, Li J, Xie X, Zhao H, Wang Y, Xu X, Zu S, Chen C, Wan D, et al. Identification of an immunogenic epitope and protective antibody against the furin cleavage site of SARS-CoV-2. *EBioMedicine* (2023) 87:104401. doi: 10.1016/j.ebiom.2022.104401

17.	Pearson WR. An Introduction to Sequence Similarity (“Homology”) Searching. *Curr Protoc Bioinformatics* (2013) 42: doi: 10.1002/0471250953.bi0301s42

18.	Fiser A, Šali A. “Modeller: Generation and Refinement of Homology-Based Protein Structure Models.,” (2003). p. 461–491 doi: 10.1016/S0076-6879(03)74020-8

19.	Shen M, Sali A. Statistical potential for assessment and prediction of protein structures. *Protein Science* (2006) 15:2507–2524. doi: 10.1110/ps.062416606

20.	Word JM, Lovell SC, Richardson JS, Richardson DC. Asparagine and glutamine: using hydrogen atom contacts in the choice of side-chain amide orientation 1 1Edited by J. Thornton. *J Mol Biol* (1999) 285:1735–1747. doi: 10.1006/jmbi.1998.2401

21.	Case DA, Cheatham TE, Darden T, Gohlke H, Luo R, Merz KM, Onufriev A, Simmerling C, Wang B, Woods RJ. The Amber biomolecular simulation programs. *J Comput Chem* (2005) 26:1668–1688. doi: 10.1002/jcc.20290

22.	Case DA, Aktulga HM, Belfon K, Cerutti DS, Cisneros GA, Cruzeiro VWD, Forouzesh N, Giese TJ, Götz AW, Gohlke H, et al. AmberTools. *J Chem Inf Model* (2023) 63:6183–6191. doi: 10.1021/acs.jcim.3c01153

23.	Jorgensen WL, Chandrasekhar J, Madura JD, Impey RW, Klein ML. Comparison of simple potential functions for simulating liquid water. *J Chem Phys* (1983) 79:926–935. doi: 10.1063/1.445869

24.	Machado MR, Pantano S. Split the Charge Difference in Two! A Rule of Thumb for Adding Proper Amounts of Ions in MD Simulations. *J Chem Theory Comput* (2020) 16:1367–1372. doi: 10.1021/acs.jctc.9b00953

25.	Joung IS, Cheatham TE. Determination of Alkali and Halide Monovalent Ion Parameters for Use in Explicitly Solvated Biomolecular Simulations. *J Phys Chem B* (2008) 112:9020–9041. doi: 10.1021/jp8001614

26.	Maier JA, Martinez C, Kasavajhala K, Wickstrom L, Hauser KE, Simmerling C. ff14SB: Improving the Accuracy of Protein Side Chain and Backbone Parameters from ff99SB. *J Chem Theory Comput* (2015) 11:3696–3713. doi: 10.1021/acs.jctc.5b00255

27.	Kirschner KN, Yongye AB, Tschampel SM, González‐Outeiriño J, Daniels CR, Foley BL, Woods RJ. GLYCAM06: A generalizable biomolecular force field. Carbohydrates. *J Comput Chem* (2008) 29:622–655. doi: 10.1002/jcc.20820

28.	Darden T, York D, Pedersen L. Particle mesh Ewald: An *N* ⋅log( *N* ) method for Ewald sums in large systems. *J Chem Phys* (1993) 98:10089–10092. doi: 10.1063/1.464397

29.	Berendsen HJC, Postma JPM, van Gunsteren WF, DiNola A, Haak JR. Molecular dynamics with coupling to an external bath. *J Chem Phys* (1984) 81:3684–3690. doi: 10.1063/1.448118

30.	Miyamoto S, Kollman PA. Settle: An analytical version of the SHAKE and RATTLE algorithm for rigid water models. *J Comput Chem* (1992) 13:952–962. doi: 10.1002/jcc.540130805

31.	Salomon-Ferrer R, Götz AW, Poole D, Le Grand S, Walker RC. Routine Microsecond Molecular Dynamics Simulations with AMBER on GPUs. 2. Explicit Solvent Particle Mesh Ewald. *J Chem Theory Comput* (2013) 9:3878–3888. doi: 10.1021/ct400314y

32.	Loncharich RJ, Brooks BR, Pastor RW. Langevin dynamics of peptides: The frictional dependence of isomerization rates of *N* ‐acetylalanyl‐ *N* ′‐methylamide. *Biopolymers* (1992) 32:523–535. doi: 10.1002/bip.360320508

33.	Peri C, Gagni P, Combi F, Gori A, Chiari M, Longhi R, Cretich M, Colombo G. Rational Epitope Design for Protein Targeting. *ACS Chem Biol* (2013) 8:397–404. doi: 10.1021/cb300487u

34.	Marchetti F, Capelli R, Rizzato F, Laio A, Colombo G. The Subtle Trade-Off between Evolutionary and Energetic Constraints in Protein–Protein Interactions. *J Phys Chem Lett* (2019) 10:1489–1497. doi: 10.1021/acs.jpclett.9b00191

35.	Serapian SA, Marchetti F, Triveri A, Morra G, Meli M, Moroni E, Sautto GA, Rasola A, Colombo G. The Answer Lies in the Energy: How Simple Atomistic Molecular Dynamics Simulations May Hold the Key to Epitope Prediction on the Fully Glycosylated SARS-CoV-2 Spike Protein. *J Phys Chem Lett* (2020) 11:8084–8093. doi: 10.1021/acs.jpclett.0c02341

36.	Scantamburlo F, Masgras I, Ciscato F, Laquatra C, Frigerio F, Cinquini F, Pavoni S, Triveri A, Frasnetti E, Serapian SA, et al. Design and Test of Molecules that Interfere with the Recognition Mechanisms between the SARS-CoV-2 Spike Protein and Its Host Cell Receptors. *J Chem Inf Model* (2024) 64:8274–8282. doi: 10.1021/acs.jcim.4c01511

37.	Onufriev A, Bashford D, Case DA. Modification of the Generalized Born Model Suitable for Macromolecules. *J Phys Chem B* (2000) 104:3712–3720. doi: 10.1021/jp994072s

38.	Weiser J, Shenkin PS, Still WC. Approximate atomic surfaces from linear combinations of pairwise overlaps (LCPO). *J Comput Chem* (1999) 20:217–230. doi: 10.1002/(SICI)1096-987X(19990130)20:2<217::AID-JCC4>3.0.CO;2-A

39.	Miller BR, McGee TD, Swails JM, Homeyer N, Gohlke H, Roitberg AE. *MMPBSA.py* : An Efficient Program for End-State Free Energy Calculations. *J Chem Theory Comput* (2012) 8:3314–3321. doi: 10.1021/ct300418h

40.	Kollman PA, Massova I, Reyes C, Kuhn B, Huo S, Chong L, Lee M, Lee T, Duan Y, Wang W, et al. Calculating Structures and Free Energies of Complex Molecules:  Combining Molecular Mechanics and Continuum Models. *Acc Chem Res* (2000) 33:889–897. doi: 10.1021/ar000033j

41.	Genoni A, Morra G, Colombo G. Identification of Domains in Protein Structures from the Analysis of Intramolecular Interactions. *J Phys Chem B* (2012) 116:3331–3343. doi: 10.1021/jp210568a
